# The genus *Hygrocybe subgenus Hygrocybe* in the Dominican Republic: phylogeny, diversity, and description of seven novel species and one variety (*Agaricomycetes*, *Hygrocybaceae*)

**DOI:** 10.3897/imafungus.17.195850

**Published:** 2026-07-31

**Authors:** Francesco Dovana, Claudio Angelini, D. Jean Lodge, Mauro Marchetti, Alfredo Vizzini

**Affiliations:** 1 Dipartimento di Bioscienze, Biotecnologie e Ambiente (DBBA), Università degli Studi di Bari “Aldo Moro”, Via Orabona 4, I-70125 Bari, Italy Department of Plant Pathology and Odum School of Ecology, University of Georgia Athens United States of America; 2 Departamento de Botánica - Jardín Botánico Nacional Jardín Botánico Nacional Dr. Rafael Ma. Moscoso, Distrito Nacional, Av. Colombia s.n., Santo Domingo, Dominican Republic Dipartimento di Bioscienze, Biotecnologie e Ambiente (DBBA), Università degli Studi di Bari “Aldo Moro” Bari Italy; 3 Via Cappuccini 78/8, I-33170 Pordenone, Italy Department of Life Sciences and Systems Biology, University of Torino Turin Italy; 4 Department of Plant Pathology and Odum School of Ecology, University of Georgia, Athens, GA 30602, United States Unaffiliated Pordenone Italy; 5 Via Molise 8, I-56123 Pisa, Italy Departamento de Botánica - Jardín Botánico Nacional Jardín Botánico Nacional Dr. Rafael Ma. Moscoso Santo Domingo Dominican Republic; 6 Department of Life Sciences and Systems Biology, University of Torino, Viale P.A. Mattioli 25, I-10125 Turin, Italy Unaffiliated Pisa Italy

**Keywords:** *
Agaricales
*, *
Agaricomycetes
*, *
Basidiomycota
*, Caribbean fungi, Neotropical diversity, nrITS-nrLSU phylogeny, taxonomy

## Abstract

The genus *Hygrocybe (Hygrocybaceae)* exhibits remarkable diversity in the tropical forests of the Dominican Republic, particularly within *H. subgenus Hygrocybe*. Following over 20 years of fieldwork (2006–2026), seven new taxa are described herein based on morphological, molecular, and ecological analyses. Phylogenetic reconstructions using nuclear ribosomal ITS and LSU sequences resolved four well-supported clades in *H. subgenus Hygrocybe* (/appalachianensis, /acutoconica, /conica, and /hypohaemacta), each corresponding to a distinct evolutionary lineage. The novel species include *H. citrinonigrescens*, distinguished by its lemon-yellow pileus, which blackens with age, and the presence of dimorphic basidia in some basidiomata; *H. jarabacoensis*, a fibrillose, greenish-yellow species associated with coniferous forests; *H. perdomoi*, a species from the Dominican Republic and Florida that features small, completely viscid, yellow-orange to scarlet-red basidiomata and shares morphological similarities with *H. acutoconica*; *H. pseudohypohaemacta* and *H. variecolor*, two phylogenetically close yet morphologically divergent taxa within *H.* section *Velosae*; and *H. xerophila*, a species described from Florida, USA, and the Dominican Republic. Molecular data also revealed cryptic diversity, such as *H. parabolica*, which is notable for its bisporic basidia and affinity for burned habitats. Dimorphic basidia and spores are reported for the first time in three species of *H. subgenus Hygrocybe* section *Hygrocybe*, viz., *H. citrinonigrescens*, *H. parabolica*, and *H. xerophila*. The study highlights the significance of the Dominican Republic as a hub of *Hygrocybe* diversity, with species exhibiting specialized adaptations to tropical ecosystems, including associations with disturbed or serpentine soils. These findings expand the understanding of Neotropical fungal biodiversity and emphasize the conservation value of Caribbean forests.

## Introduction

Among the waxcap fungi in the *Hygrocybaceae* (Padamsee & Lodge) Vizzini, Consiglio & P. Alvarado, recently segregated from the *Hygrophoraceae* Lotsy (Vizzini et al. 2024), the worldwide genus *Hygrocybe* (Fr.) P. Kumm. includes approximately 120 described species (He et al. 2019), but many species remain undescribed. While in Europe these fungi are primarily found in nutrient-poor grasslands and pastures, in other regions of the world, such as the tropical and boreal areas of America, they are predominantly distributed in forest ecosystems (Lodge et al. 2014). Previous studies that focused on the biodiversity of *Hygrophoraceae* s.l. (*Hygrophorineae* Aime, Dentinger & Gaya, *Agaricales* Underw.) in the Greater Antilles reported the presence of at least 18 taxa belonging to the genus *Hygrocybe* (Fr.) P. Kumm., of which 12 species were described as new between 2000 and 2004 (Cantrell and Lodge 2000, 2001, 2004). To date, five taxa have been reported from the Dominican Republic (Cantrell and Lodge 2000, 2004; Lodge et al. 2014): *H. cantharellus* (Schwein.) Murrill, *H. coccinea* (Schaeff.) P. Kumm., and *H. viridiphylla* Lodge, S.A. Cantrell & T.J. Baroni in section *Coccineae* Fayod within subgenus *Pseudohygrocybe* Bon (Cantrell and Lodge 2004), H. conica var. brevispora (Dennis) S.A. Cantrell & Lodge in section *Hygrocybe* (Cantrell and Lodge 2000), and *H. aff. hypohaemacta* in section *Velosae* Lodge, Ovrebo & Padamsee (Lodge et al. 2014), the latter two belonging to subgenus *Hygrocybe*. Over the course of 20 years of research in the Dominican Republic (from 2006 to 2026) conducted by one of the authors (C.A.), seven species of *Hygrocybe subgenus Hygrocybe* were recognized. Macromorphologically, *Hygrocybe subgenus Hygrocybe* is characterized by its pileus, which typically exhibits shades of red, orange, yellow, green, or purple because of DOPA-derived betalain pigments. In rare instances, the pileus may appear colorless or take on a fuscous hue as it ages or when bruised, resulting from DOPA transformation. Fibrous or glutinous partial veils are only occasionally observed. The lamellae are primarily free or narrowly attached to the stipe, with broad attachment occurring only occasionally through a decurrent tooth. Micromorphologically, the lamellar trama hyphae are arranged in a strictly parallel formation, typically feature tapered ends, and range in length from over 140 μm to more than 1,000 μm (Kühner 1976, 1980; Kovalenko 1989; Arnolds 1990; Bon 1990; Candusso 1997; Young 2005; Boertmann 2010, 2012; Lodge et al. 2014; Wang et al. 2020). Unless the basidia and spores display dimorphism, the basidia are generally three to five times longer than their corresponding basidiospores, in contrast to those in *H. subgenus Pseudohygrocybe*, where the basidia exceed five times the length of the basidiospores (Lodge et al. 2014). The main aims of this study are (1) to present preliminary data on the biodiversity of the genus *Hygrocybe*, subgenus *Hygrocybe*, in the Dominican Republic and (2) to describe seven new taxa based on both morphological and molecular analyses.

## Materials and methods

### Morphology

Macroscopic descriptions were based on observations of fresh basidiomata. Detailed field notes on the fresh basidiomata were supplemented by macroscopic descriptions and ecological details, including habitat, the timing of fruiting, and associated plant communities. Collected samples were photographed in their natural environment using a Nikon Coolpix 8400 digital camera. The micromorphological characters were analyzed from dried samples using various mounting media, including water; L4 (7.2 g KOH, 160 mL glycerol, 840 mL dH_2_O, 7.6 g NaCl, and 5 mL Invadin) (Clémençon 1972); Melzer’s reagent; ammoniacal Congo red; phloxine; and cotton blue. Dried fragments were rehydrated with water and then mounted in L4. All microscopic measurements were conducted using oil immersion at a magnification of 1,000× with a Nikon Eclipse 80i microscope. Basidiospore measurements were obtained from captured images of all spores present in a single microscope field of view. The spores were taken from lamellar squash mounts of rehydrated, dried material from mature specimens and measured using the Nikon DS-L1 camera control unit. Spore and basidium dimensions are expressed as (a) b–*c*–d(e), where (a) = minimum value, b = average − standard deviation, *c* = average, d = average + standard deviation, and (e) = maximum value. The notation [*n/m/p*] indicates that measurements were made on *n* randomly selected spores or basidia from *m* basidiomata of *p* collections. At least 30 spores were measured for every collection. The letter *Q* represents the ratio of the length to the width of the spores in side view or of the basidia. The approximate spore and basidium volume (*V*, in μm^3^) was calculated as that of a prolate ellipsoid (Gross 1972; Meerts 1999; Clémençon 2004) and was provided with the same statistics as the other spore and basidium parameters. Photographs of microscopic features were taken using a Nikon DS-5M digital camera with a preset resolution of 2,560 × 1,980 pixels connected to a Nikon Eclipse 80i optical microscope with both bright-field and interference-contrast optics and were saved in TIFF format. They were then processed using Adobe Photoshop CC 2019, version 20.0.10. In some cases, the background color of the photograph was replaced with an alternative uniform background using the Magic Wand tool with appropriate tolerance settings to highlight the morphology of the microscopic characters while maintaining their original size and hue. In other cases, the best individual spores from microscopy photographs were copied using the Polygonal Lasso tool or, alternatively, the Magnetic Lasso tool in Adobe Photoshop CC 2019, version 20.0.10, onto a new monochrome sheet with the same resolution (2,560 × 1,980 pixels) to maintain the original spore size and colors. Names of taxa and nomenclatural author citations follow Index Fungorum (2026). Studied specimens are deposited in the Fungarium of the Jardín Botánico Nacional Dr. Rafael M. Moscoso, Dominican Republic (JBSD); the Center for Forest Mycology Research herbarium and culture collection, USDA, Madison, Wisconsin, USA (CFMR); the University of Florida Herbarium, USA (FLAS) (Thiers 2026); the Kriebel Herbarium, Purdue University, West Lafayette, Indiana, USA (PUL); the iNaturalist Herbarium; and the authors’ private fungaria “ANGE” and “DJL,” maintained by Claudio Angelini and D. Jean Lodge, respectively.

### DNA extraction, PCR amplification, and DNA sequencing

Genomic DNA was extracted from dried fragments of 20 collected specimens using the CTAB procedure (Doyle and Doyle 1987). The internal transcribed spacer (nrITS) was amplified with primers ITS1F (Doyle and Doyle 1987; Gardes and Bruns 1993) and ITS4 (White et al. 1990) or with ITS1F/ITS2 (White et al. 1990; Gardes and Bruns 1993) for the ITS1 region and ITS3/ITS4 (White et al. 1990) for the ITS2 region. A portion of the nuclear ribosomal large subunit ribosomal RNA (nrLSU) was amplified with primers LR0R/LR7 (Vilgalys and Hester 1990). The PCR products were purified and sequenced according to the Sanger method using the same primers as those used for amplification.

### Sequence alignment, dataset assembly, and phylogenetic analysis

The sequences obtained in this study were checked and assembled using Geneious version R11.1.5 (Kearse et al. 2012) and compared with those available in the GenBank database (https://www.ncbi.nlm.nih.gov/genbank/) (accessed 25 May 2025) using the BLASTN algorithm (Altschul et al. 1990). The accession numbers and origins of the sequences used in the phylogeny are reported in Table 1. The nrITS and nrLSU datasets include sequences of *Hygrocybe subgenus Hygrocybe* that were treated in previous phylogenetic analyses (Lodge et al. 2014; Latha and Manimohan 2018; Wang et al. 2019, 2020; Latha et al. 2023, 2024). *Hygroaster albellus* Singer CFMR-PR6377 and *Hygroaster* sp., identified as *H. nodulisporus* (Dennis) Ralaiv., Niskanen & Liimat., CFMR-PR6378, were used as outgroup taxa. The nrITS and nrLSU sequences were aligned independently using MAFFT version 7.017 with the E-INS-i algorithm (Katoh and Standley 2013). The sequences were then concatenated using Geneious version R11.1.5. To create a phylogenetic tree for the various taxa studied, maximum likelihood (ML) analysis was conducted using IQ-TREE 2 (Minh et al. 2020). The optimal models were identified using ModelFinder (Kalyaanamoorthy et al. 2017). The reliability of the clades was evaluated using the SH-like approximate likelihood-ratio test (SH-aLRT), with 1,000 replicates (Guindon et al. 2010), and the ultrafast bootstrap approximation (UFB), also with 1,000 replicates (Hoang et al. 2018). Bayesian inference (BI) was performed using MrBayes version 3.2 (Minh et al. 2020; Ronquist et al. 2012) via the CIPRES Science Gateway (Miller et al. 2010). Partition Finder 2 (Lanfear et al. 2016) was used to determine the best partitioning strategies and evolutionary models for the nrITS and nrLSU subsets within the MrBayes framework. The GTR+G+I model was chosen for each partition, and two independent analyses with four MCMC chains were conducted over 10 million generations, with trees sampled every 1,000 generations. The initial 25% of trees were excluded as burn-in, and a majority-rule consensus tree was computed from the remaining trees to derive estimates of Bayesian posterior probabilities (BPP). Significant support was defined as ≥80% for the SH-aLRT, ≥95% for UFB in the ML analysis, and ≥0.95 for BPP in the BI analysis.

**Table 1.** Taxa, vouchers, and GenBank accession numbers used in the molecular analyses of nrITS and nrLSU sequences. Newly generated sequences are shown in bold.

**Name of the species**	**Voucher no**.	**Country**	**nrITS**	**nrLSU**	**Reference(s)**
Hygrocybe acutoconica f. japonica	CFMR JP-2	Japan	KF291161	KF291162	Lodge et al. (2014)
*Hygrocybe acutoconica var. pallidocarnea*	SIENA_5495 (holotype)	Italy	PP965547	PP965550	Siquier et al. (unpublished); Siquier et al. (2024)
*Hygrocybe acutoconica var. persistens*	H1	Hungary	FM208852	–	Babos et al. (2011)
*Hygrocybe aff. citrinovirens*	DJL05TN10 (TENN)	USA (Tennessee)	KF291090	KF291091	Lodge et al. (2014)
*Hygrocybe aff. conica*	PBM 918/AFTOL-ID 729	USA	AY854074	AY684167	Matheny and Hibbett (unpublished); Matheny et al. (2006)
*Hygrocybe aff. hypohaemacta*	CFMR BZ-1903	Belize	EU435150	EU435150	Lodge and Ovrebo (2008)
*Hygrocybe aff. hypohaemacta*	RC-GUY-12-062	French Guiana	MF038931	–	Jaouen et al. (2019)
*Hygrocybe aff. hypohaemacta*	iNat63858864	USA (Florida)	OP028520	–	Franck et al. (unpublished)
*Hygrocybe aff. neofirma*	CFMR BZ-1926	Belize	–	KF291166	Lodge et al. (2014)
*Hygrocybe aff. prieta*	CFMR BZ-3079	Belize	KF291168	KF291169	Lodge et al. (2014)
*Hygrocybe appalachianensis*	TENN:61606	USA (Tennesseee)	FJ596847	–	Hughes et al. (2009)
*Hygrocybe cf. flavescens*	MushroomObserver.org/308214	USA (California)	MG926551	MG926551	Rockefeller (unpublished)
*Hygrocybe cf. hypohaemacta*	RLC_272_iNat_101672217	Ecuador	OQ871701	–	Vandegrift et al. (2023)
*Hygrocybe chloochlora*	SAC-PR-9899 (CFMR PR-5350)	Puerto Rico	KF381522	–	Lodge et al. (2014)
*Hygrocybe chlorophana*	CFMR Boertmann 2002/9	Denmark	EU435148	EU435148	Lodge et al. (2014)
***Hygrocybe citrinonigrescens***	**JBSD 141871 (holotype)**	**Dominican Republic**	**PZ148591**	**–**	**This study**
***Hygrocybe citrinonigrescens***	**ANGE826**	**Dominican Republic**	**PZ148593**	**–**	**This study**
***Hygrocybe citrinonigrescens***	**ANGE817**	**Dominican Republic**	**PZ148600**	**–**	**This study**
*Hygrocybe citrinovirens*	H2	Hungary	FM208853	–	Babos et al. (2011)
*Hygrocybe conica*	1499	Italy	JF908057	–	Osmundson et al. (2013)
*Hygrocybe conica*	HMJAU61878	China	ON045774	ON046286	Qq (unpublished)
*Hygrocybe conica var. conica*	H31	Hungary	FM208878	–	Babos et al. (2011)
*Hygrocybe conica var. conicoides*	QHU20235	China	OM970939	OM942720	Bai and Qiang (unpublished)
*Hygrocybe debilipes*	GDGM59314 (holotype)	China	MW001783	MW007877	Wang et al. (2020)
*Hygrocybe glutinipes*	RBG Kew K(M)126644	UK	EU784309	–	Brock et al. (2009)
*Hygrocybe glutinipes var. rubra*	CFMR NC-9	USA (North Carolina)	EU435149	EU435149	Lodge et al. (2014)
*Hygrocybe griseonigricans*	GDGM73527 (holotype)	China	MW001790	MW007883	Wang et al. (2020)
*Hygrocybe helobia*	14600	Italy	JF908056	–	Osmundson et al. (2013)
*Hygrocybe helobia var. mollis*	RBG Kew K(M)62585	UK	EU784330	–	Brock et al. (2009)
*Hygrocybe hypohaemacta*	KUM61079 (CFMR)	Malaysia	–	KF381551	Lodge et al. (2014)
*Hygrocybe indica*	CAL 1726 (holotype)	India	MH938535	MH938534	Latha and Manimohan (2018)
*Hygrocybe intermedia*	RBG Kew K(M)126648	UK	EU784321	–	Brock et al. (2009)
***Hygrocybe jarabacoensis***	**JBSD 141872 (holotype)**	**Dominican Republic**	**PZ148590**	**PZ145851**	**This study**
***Hygrocybe jarabacoensis***	**ANGE888**	**Dominican Republic**	**PZ148595**	**PZ145849**	**This study**
***Hygrocybe jarabacoensis***	**ANGE1940**	**Dominican Republic**	**PZ148596**	**PZ145850**	**This study**
*Hygrocybe konradii var. konradii*	4725	Italy	JF908059	–	Osmundson et al. (2013)
*Hygrocybe konradii var. konradii*	Boertmann 2004/6	Denmark	KF306329	KF306330	Lodge et al. (2014)
*Hygrocybe nigrescens var. brevispora*	SAC-0005 (CFMR PR-5964)	Puerto Rico	KF291130	KF291131	Lodge et al. (2014)
*Hygrocybe nigrescens var. brevispora*	TWH1029 (BPI 925554)	Brazil	PP830807	–	Jacobsen et al. (unpublished); Jacobsen et al. (2025)
*Hygrocybe nigrescens var. brevispora*	ECO-TA-HO 7868	Mexico	MF156250	–	Arias-Hernandez et al. (unpublished)
*Hygrocybe noninquinans*	PR-6372 (DJL-PR-1)	Puerto Rico	KF291127	KF291129	Lodge et al. (2014)
*Hygrocybe occidentalis var. scarletina*	PR-6493 (Cancerel PR 02)	Puerto Rico	EU435151	EU435151	Lodge et al. (2014)
***Hygrocybe parabolica***	**JBSD 141877 (holotype)**	**Dominican Republic**	**PZ148587**	**–**	**This study**
*Hygrocybe pellucida*	DKP-SERB131 (holotype)	India	OR982363	OR982364	Latha et al. (2024)
***Hygrocybe perdomoi***	**JBSD 141873 (holotype)**	**Dominican Republic**	**PZ148589**	**PZ145852**	**This study**
***Hygrocybe perdomoi***	**ANGE816**	**Dominican Republic**	**PZ148601**	**–**	**This study**
***Hygrocybe perdomoi***	**ANGE815**	**Dominican Republic**	**PZ148592**	**–**	**This study**
***Hygrocybe perdomoi***	**ANGE818**	**Dominican Republic**	**PZ148602**	**–**	**This study**
*Hygrocybe persistens*	H23	Hungary	FM208872	–	Babos et al. (2011)
*Hygrocybe pseudoacutoconica*	GDGM57214 (holotype)	China	MH844889	NG_067838	Wang et al. (2019)
*Hygrocybe pseudoconica*	5159	Italy	JF908060	–	Osmundson et al. (2013)
***Hygrocybe pseudohypohaemacta***	**JBSD 141874 (ANGE823)**	**Dominican Republic**	**PZ281543 (ITS1), PZ281551 (ITS2)**	**PZ145855**	**This study**
***Hygrocybe pseudohypohaemacta***	**JBSD 141878 (holotype)**	**Dominican Republic**	**PZ148603**	**PZ145854**	**This study**
***Hygrocybe pseudohypohaemacta***	**ANGE 692**	**Dominican Republic**	**PZ148597**	**–**	**This study**
***Hygrocybe pseudohypohaemacta***	**ANGE825**	**Dominican Republic**	**PZ281542 (ITS1), PZ281550 (ITS2)**	**–**	**This study**
***Hygrocybe pseudohypohaemacta***	**ANGE824**	**Dominican Republic**	**PZ148598**	**–**	**This study**
***Hygrocybe pseudohypohaemacta***	**ANGE822**	**Dominican Republic**	**PZ148594**	**–**	**This study**
*Hygrocybe roseopallida*	PMA Ovrebo-4073	Panama	EU435152	EU435152	Lodge and Ovrebo (2008)
*Hygrocybe rubroconica*	GDGM45213 (holotype)	China	MW001786	MW007881	Wang et al. (2020)
*Hygrocybe singeri*	TENN:023666 (isotype)	USA (Oregon)	HQ185708	–	Matheny and Wolfenbarger, (unpublished)
*Hygrocybe snigdha*	DKP-SERB95 (holotype)	India	OR125065	OR127148	Latha et al. (2023)
***Hygrocybe* sp**.	**ANGE436**	**Dominican Republic**	**PZ148599**	**PZ145853**	**This study**
*Hygrocybe* sp.	ARF4859/FLAS-F-70002	USA (Florida)	OP580224	–	Smith et al. (unpublished)
*Hygrocybe* sp.	FLAS-F-61222	USA (Florida)	MH211824	–	Kaminsky et al. (unpublished)
*Hygrocybe* sp.	FLAS-F-68949	USA (Florida)	OP163190	–	Kaminsky and Smith (unpublished)
*Hygrocybe* sp. aff. *H. chloochlora*	CFMR BZ-3042	Belize	EU435147	EU435147	Lodge and Ovrebo (2008)
*Hygrocybe* sp. ‘IN10’	OMDL iNat # 195601387	USA (Florida)	PP791118	–	Ostuni et al. (unpublished)
*Hygrocybe* sp. ‘IN10’	OMDL iNat # 195601389	USA (Florida)	PP850544	–	Ostuni et al. (unpublished)
*Hygrocybe* sp. ‘IN10’	S.D. Russell ONT Mushroom Observer 501267	USA (Florida)	PP383936	–	Russell (unpublished)
*Hygrocybe spadicea*	Griffith-Wauenfawr 25-Oct-2006 (ABS)	UK	KF291201	–	Lodge et al. (2014)
*Hygrocybe subpapillata*	RBG Kew K(M)54960	UK	EU784350	–	Brock et al. (2009)
***Hygrocybe variecolor***	**JBSD 141875 (holotype)**	**Dominican Republic**	**PZ148612 (ITS1), PZ221056 (ITS2)**	**PZ145856**	**This study**
***Hygrocybe xerophila var. floridana***	**FLAS-F-61718 (FLAS) (holotype)**	**USA (Florida)**	**MH212066**	**–**	**This study**
***Hygrocybe xerophila var. xerophila***	**JBSD 141876 (holotype)**	**Dominican Republic**	**PZ148588**	**–**	**This study**
Uncultured soil fungus	clone 164-14	USA (Minnesota)	DQ420708	**–**	Waldrop et al. (2006)
**OUTGROUP TAXA**					
*Hygroaster albellus*	CFMR PR-6377 (AFTOL-1997)	Puerto Rico	KF381521	EF551314	Lodge et al. (2014)
*Hygroaster* sp. as *H. nodulisporus*	CFMR PR-6378 (AFTOL ID 2020)	Puerto Rico	**–**	EF561625	Lodge et al. (2014)

### Molecular results

**Figure 1.**
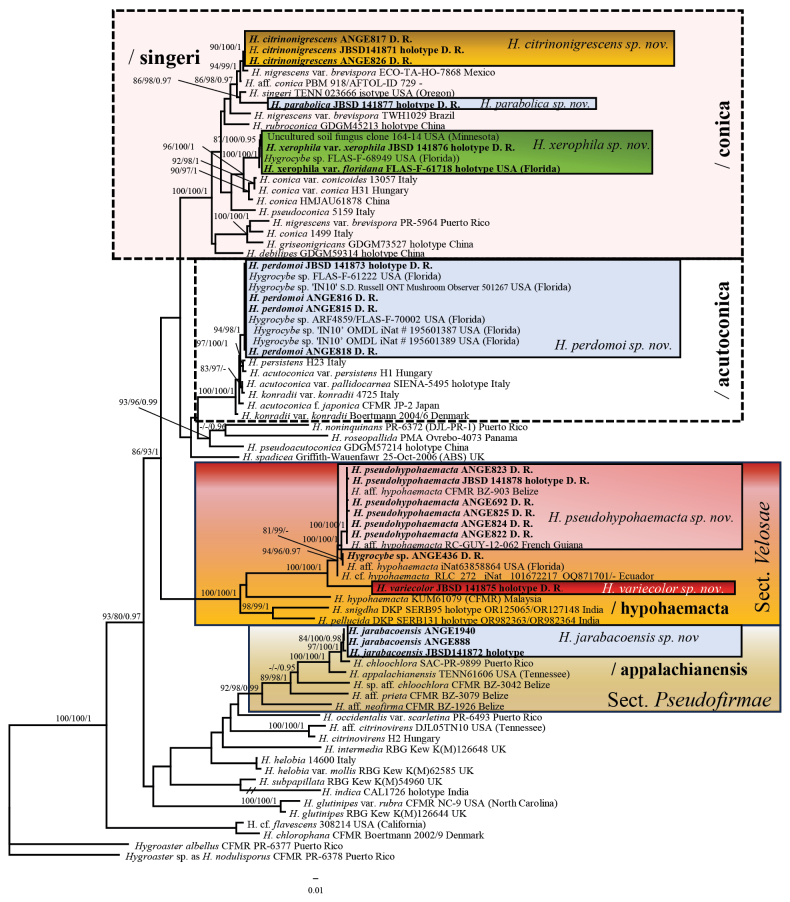
Phylogenetic tree generated by maximum likelihood analysis using the concatenated nrITS and nrLSU sequences of the genus *Hygrocybe subgenus Hygrocybe*. *Hygroaster albellus* and *H. nodulisporus* were used as outgroup taxa. Values above or below the branches indicate SH-aLRT support ≥80%/UFB ≥95%/BPP ≥0.95. Newly generated sequences are highlighted in bold. D.C. = Dominican Republic.

A total of 20 nrITS and 8 nrLSU sequences were newly generated for this study (Table 1). Both ML and BI analyses produced the same topology; therefore, only the ML tree, with SH-aLRT, UFB, and BPP values, is shown (Fig. 1). The combined nrITS and nrLSU data matrix comprised 77 sequences and 1,838 characters.

In the phylogenetic reconstruction, four well-supported main clades were identified within *H. subg. Hygrocybe*:

- /acutoconica: includes the novel species *H. perdomoi*;
- /appalachianensis: includes species belonging to *H.* sect. *Pseudofirmae* Lodge & Padamsee, including the novel species *H. jarabacoensis*;
- /conica: includes the novel species *H. citrinonigrescens*, *H. parabolica*, *H. xerophila*, and its novel variety *floridana*;
- /hypohaemacta: includes species belonging to *H.* section *Velosae*, specifically the novel species *H. pseudohypohaemacta* and *H. variecolor*.

## Taxonomy

### Hygrocybe citrinonigrescens

Taxon classificationFungiAgaricalesHygrophoraceae

Angelini, Dovana, Lodge, M. Marchetti & Vizzini sp. nov.

64C4FB6C-4AAE-5F90-AC51-2BD638862F2F

864517

Figs 2, 3, 4

#### Etymology.

*citrino* refers to the lemon-yellow pileus, while *nigrescens* denotes the entirely blackening basidioma.

**Figure 2.**
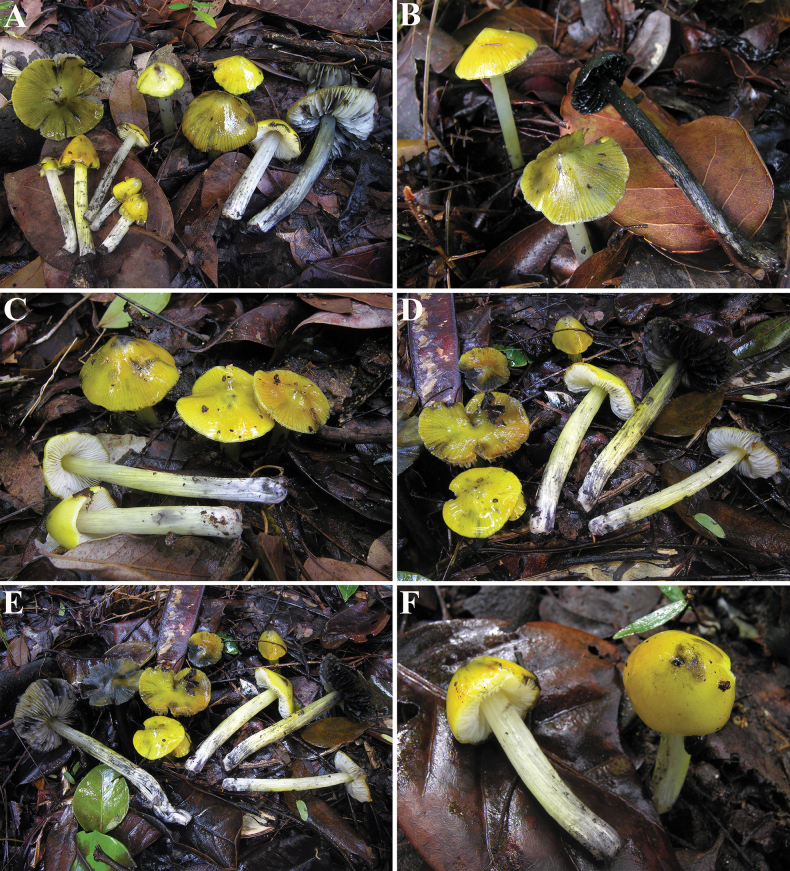
*Hygrocybe citrinonigrescens*, fresh basidiomata. **A–C**JBSD 141871, holotype; **D–E** ANGE817; **F** ANGE826. Photos by C. Angelini.

**Figure 3.**
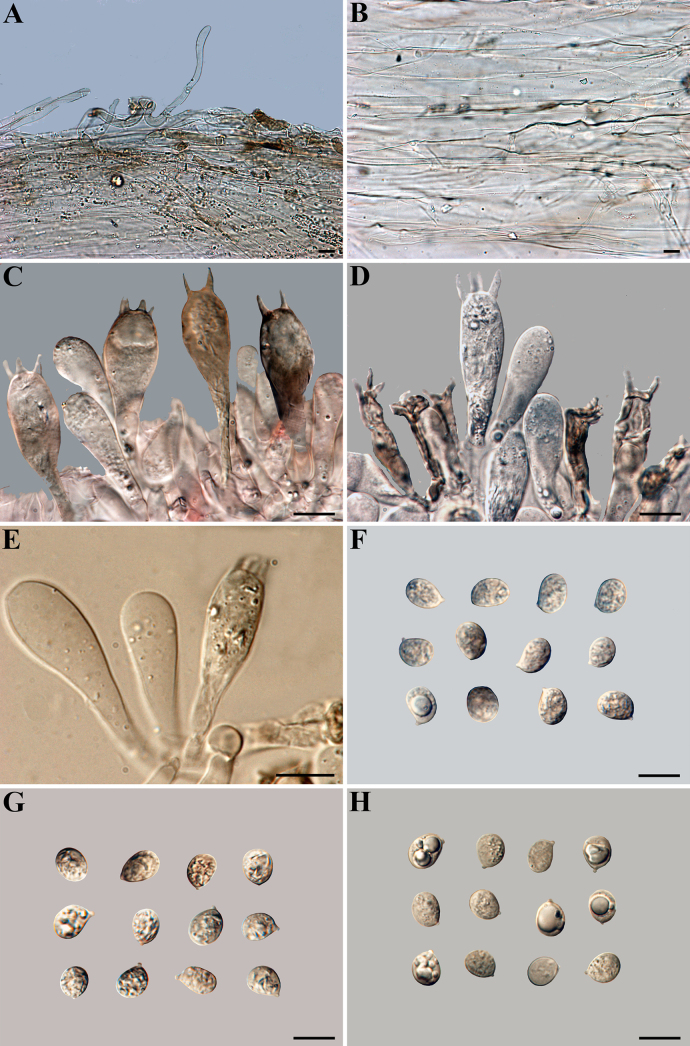
*Hygrocybe citrinonigrescens*, microscopic features. **A** Pileipellis; **B** subcutis; **C** basidia filled with brown necropigment (necrobasidia); **D** basidia (some mature, others collapsed and necropigmented); **E** basidium and basidiola; **F–H** basidiospores. (**A**, **D**, **F**) JBSD 141871, holotype; (**B–C**, **E**, **G**) ANGE817; (**H**) ANGE826. **A–H** in ammoniacal Congo red. Photos by M. Marchetti. Scale bars: 10 μm.

**Figure 4.**
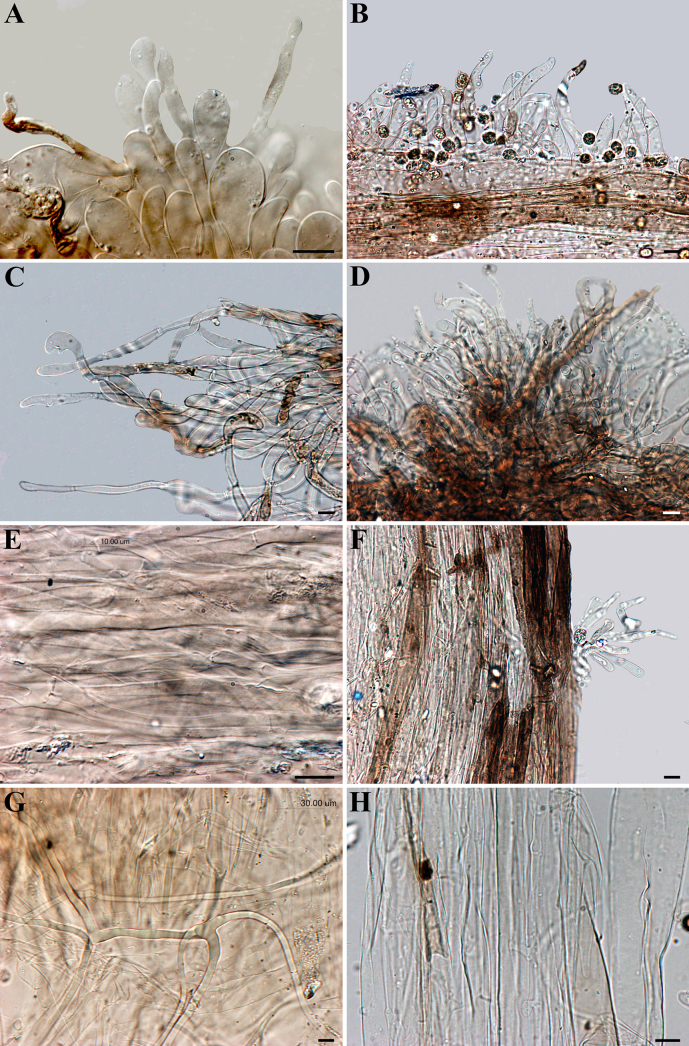
*Hygrocybe citrinonigrescens*, microscopic features. **A** Lamellar edge with pseudocystidia (ANGE817); **B–D** caulocystidia present in the apical portion of the stipe (ANGE817, JBSD 141871, holotype, ANGE826); **E** elements of the hymenophoral trama (ANGE817); **F** stipitipellis from the mid-stipe, showing caulocystidia (ANGE817); **G** subcutis (ANGE826); **H** stipititrama (ANGE817). **A–H** in ammoniacal Congo red. Photos by M. Marchetti.Scale bars: 10 μm.

#### Diagnosis.

Differing from *H. singeri* in having a vivid lemon-yellow pileus, shorter basidiospores, and divergence in ribosomal sequences; differing from H. conica var. brevispora in lacking red coloration in the pileus and stipe and in its divergence in ribosomal sequences.

#### Types.

Dominican Republic, Puerto Plata, Sosúa, loc. Cemetery 19.745998, –70.539411, 11 December 2016, C. Angelini, JBSD 141871 Holotype (ANGE814 Isotype). GenBank: ITS = PZ148591.

#### Description.

***Pileus*** 15–40 mm diam., conical at first, later expanded to applanate with a ± distinct, usually acute umbo but sometimes broadly conical, margin not or very slightly lobed, translucent-striate up to more than half the radius; surface smooth, slightly fibrillose, strongly glutinous, lemon-yellow, often flushed with orange, strongly blackening with age. ***Lamellae*** subfree, ventricose, up to 7 mm broad, intercalated with lamellulae, white, thin, and moderately distant, with irregular edge, blackening when handled or with age. ***Stipe*** 35–70 × 7–12 mm, cylindrical and straight, tapered towards the apex, light lemon-yellow, whitish at the base; surface viscid, distinctly fibrillose-ribbed and twisted, blackening when handled or with age, fistulose. All parts ± blackening when handled or with age and weathered or old basidiomata may be entirely black. ***Context*** very thin in the pileus (1–1.5 mm) white-orange, thicker in the stipe (2–3 mm), whitish silky-watery, blackening. ***Odor*** and ***taste*** indistinct.

***Basidiospores*** (7.5–)8.5–*9.2*–9.9(–11.0) × (6.0–)6.8–*7.4*–7.9(–8.5) µm [153/5/3], *Q* =1.15–*1.25*–1.36, *V* = 214–*264*–314 µm^3^, broadly ellipsoid to ellipsoid, varying seamlessly from small to large (without a clearcut distinction between microspores and macrospores), often with a subplane adaxial side, hyaline, smooth, thin-walled, with a large and obtuse hilar appendix, multiguttulate, with greenish guttules observed in bright-field microscopy. ***Basidia*** mainly monomorphic (as *macrobasidia*), 40–45.8(–55.0) × (9.9–)11.2–12.4(–14.8) µm [42/4/3], *Q* = 2.85–*3.30*–3.75, *V* = 2443–*3326*–4210 µm^3^, broadly clavate, usually with a long tapering and narrow peduncle, tetrasporic to bisporic, often with guttular contents, hyaline, with blackish-brown contents when old (necropigments), sterigmata up to 6(–7) µm long;); *microbasidia* very rare, maturing before macrobasidia, many partly collapsed, cylindrical-clavate, (25–)27–31 × (5–)7–8.5(–10.5) µm. ***Basidiola*** broadly clavate, hyaline, then with blackish-brown cytoplasmic contents. ***Lamellar edge*** fertile. ***Pseudocystidia*** 25–35 × 4–6(–7) µm, rare, subcylindrical, sometimes sinuous, often subacute apically, sometimes with an apical appendage. ***Hymenophoral trama*** regular, consisting of more or less parallel hyphae, subcylindrical to subfusiform, some over 200 µm long, 3–10 µm wide. ***Pileipellis*** a gelatinized cutis, with mainly radial, repent hyphae, cylindrical, frequently exceeding 200 µm in length, 3–10 µm wide, subhyaline, then blackish-brown when old; some ascending pileocystidioid elements present, 2–6 µm wide. ***Subcutis*** regular, consisting of parallel, subcylindrical hyphae, frequently exceeding 200 µm in length, 15–30 µm wide, hyaline then blackish-brown when old. ***Stipitipellis*** a slightly gelatinized cutis, consisting of parallel hyphae, subcylindrical, frequently exceeding 200 µm in length, 3–10 μm wide, thin-walled to slightly thick-walled (wall up to 1 μm thick), subhyaline, then blackish-brown when old. **Caulocystidia** present especially on stipe apex, 30–100 × 5–15 µm, versiform, subcylindrical, sinuous, bilobed, clavate, hyaline when young, sometimes with scattered guttular deposits, refractive crystalline deposits abundant. ***Stipititrama*** regular, of parallel subcylindrical elements, often over 200 µm long, 10–20 µm wide, hyaline then blackish-brown when old. ***Clamp-connections*** present throughout.

#### Habit, habitat, occurrence, and distribution.

Gregarious, in groups of a few to many basidiomata, on litter in broad-leaved forests a few kilometers from the sea. Common but localized. Thus far known only from the Dominican Republic.

#### Additional specimens examined.

Dominican Republic, Puerto Plata, Sosúa, loc. Cemetery 19.745998, –70.539411, 22 December 2016, C. Angelini (ANGE817); *ibidem*, 25 December 2016 (ANGE826).

#### Notes.

*Hygrocybe citrinonigrescens* is characterized by its medium-small size, entirely slimy basidiomata, a translucent-striate pileus margin, vivid lemon-yellow colors of the pileus, a ribbed and twisted stipe, and strong blackening of all parts of the basidiomata. The three sequences of *H. citrinonigrescens* form a well-supported clade (SH-aLRT = 90, UFB = 100, BPP = 1) within the /singeri clade (SH-aLRT = 86, UFB = 98, BPP = 0.97). The /singeri clade includes the isotype of *H. singeri* from Washington State (TENN023666), a sequence deposited as *H. aff. conica* (AFTOL_ID_729), a sequence deposited as *H. nigrescens var. brevispora* from Mexico (ECO-TA-HO 7868), and the novel species *H. parabolica*. The morphologically similar, blackening *Hygrocybe conica var. peradenyca* (Sacc.) Pegler was described from Sri Lanka but differs in having yellow vs. white lamellae, a non-slimy pileus and stipe, and narrower spores, 7–10 × 5–6 µm (Dennis 1970; Pegler 1986). *H. atrosquamosa* Pegler from Martinique and Tobago usually has a yellow, blackening pileus and spores similar in size and shape but differs from *H. citrinonigrescens* in having a strongly fibrillose, non-viscid pileus surface (Pegler 1983). Among the species phylogenetically closest to *H. citrinonigrescens*, *H. nigrescens var. brevispora* (Dennis) Pegler, recently recombined as H. conica var. brevispora (Dennis) S.A. Cantrell & Lodge, is easily distinguished macroscopically, as it has reddish coloration on both the pileus and the stipe when young (Dennis 1970; Lodge and Pegler 1990; Cantrell and Lodge 2000). Collections identified as H. conica var. brevispora are polyphyletic (Fig. 1); the species was described by Dennis as *Hygrophorus nigrescens var. brevisporus* from Venezuela. *Hygrocybe singeri* (A.H. Sm. & Hesler) Singer, described from northwestern North America as *Hygrophorus singeri* A.H. Sm. & Hesler, can be morphologically distinguished from *H. citrinonigrescens* by its pileus color, which ranges from red-orange to yellow rather than vivid lemon-yellow, and by its longer, elliptical basidiospores (9–12 × 5–6 μm) (Smith and Hesler 1954).

### Hygrocybe jarabacoensis

Taxon classificationFungiAgaricalesHygrophoraceae

Angelini, Dovana, Lodge, M. Marchetti & Vizzini sp. nov.

CBC59BC3-165E-590B-A8F3-B425A42B55E0

864518

Figs 5, 6

#### Etymology.

The specific epithet *jarabacoensis* refers to the type locality, Jarabacoa, a town in the Central Mountain Range of the Dominican Republic where the species was collected.

**Figure 5.**
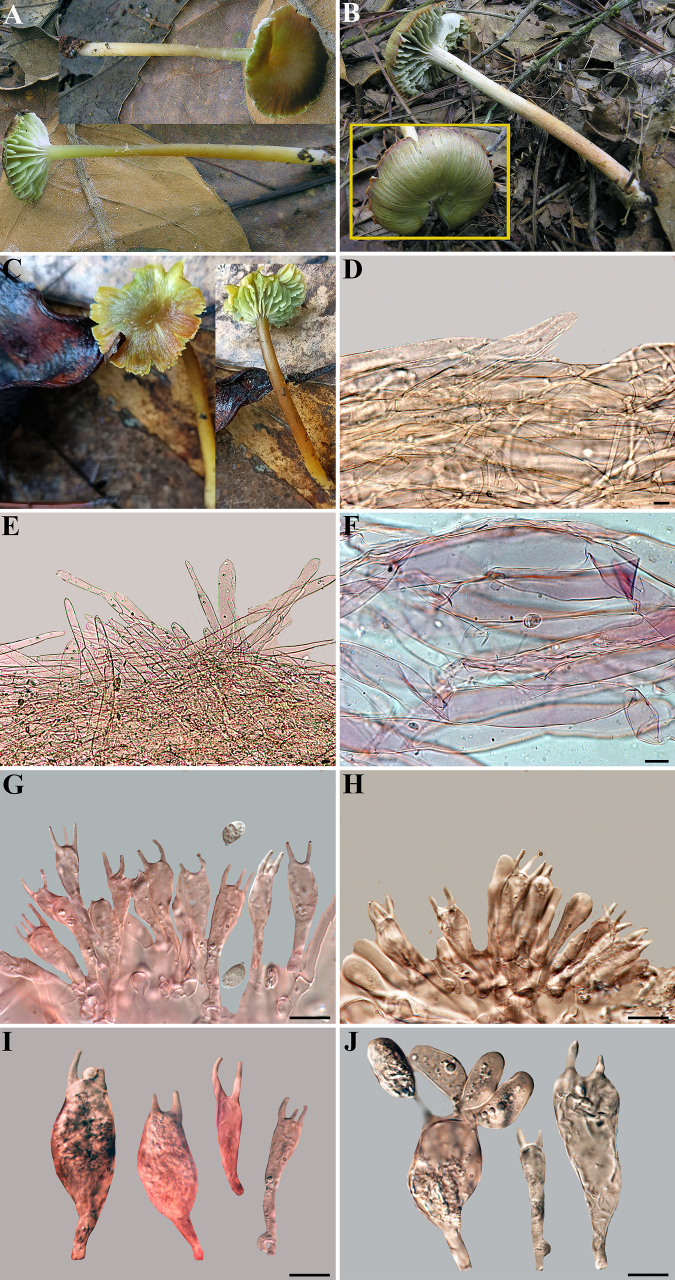
*Hygrocybe jarabacoensis*. **A–C** Fresh basidiomata (ANGE888, JBSD 141872, holotype, ANGE1940); **D–E** pileipellis (JBSD 141872, holotype, ANGE1940); **F** elements of the hymenophoral trama (JBSD 141872, holotype); **G–H** microbasidia (JBSD 141872, holotype, ANGE1940); **I** macrobasidia and microbasidia (JBSD 141872, holotype); **J** macrobasidia with macrospores and a microbasidium (ANGE1940). **D–E**, **G–J** in ammoniacal Congo red; **F** in phloxine. Photos by C. Angelini (**A–C**) and M. Marchetti (**D–J**). Scale bars: 10 μm.

**Figure 6.**
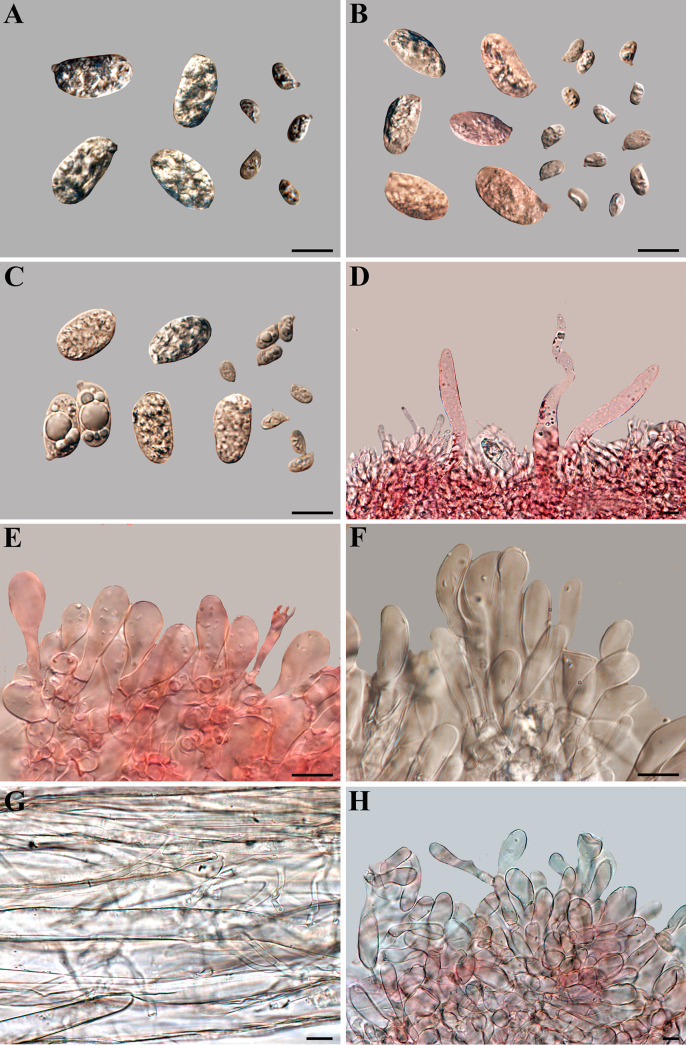
*Hygrocybe jarabacoensis*, microscopic features. **A–C** Macrospores (left) and microspores (right) (ANGE888, JBSD 141872, holotype, ANGE1940); **D** pseudocystidia (JBSD 141872, holotype); **E–F** cheilocystidia (JBSD 141872, holotype, ANGE888); **G** stipititrama (JBSD 141872, holotype); **H** caulocystidia (JBSD 141872, holotype). **A–H** in ammoniacal Congo red. Photos by M. Marchetti. Scale bars: 10 μm.

#### Diagnosis.

Differing from *H. chloochlora* in the absence of a membranous marginal fringe and a perforation on the pileus and divergence in ribosomal sequences.

#### Types.

Dominican Republic, La Vega, Jarabacoa 19.188667, –70.592655, 7 March 2020, C. Angelini, JBSD 141872 Holotype (ANGE1374 Isotype). GenBank: ITS = PZ148590, LSU = PZ145851.

#### Description.

***Pileus*** 25–40 mm diam., convex then plane, sometimes becoming depressed but not perforated at center, margin not lobed and not striate; surface dry or moist but not viscid, radially fibrillose-rugose and cracked in age, strongly rimose if dry, yellow-green, reddish(brownish)-green. ***Lamellae*** adnate-emarginate or decurrent with a tooth, ventricose, up to 7 mm broad, intercalated with 1–2 lengths of lamellulae, olive green, yellow-green, thick, and distant, with pale, irregular eroded edge. ***Stipe*** 45–75 × 2–5 mm, elongate, straight and ± cylindrical, greenish white towards the apex, tinted pale red or yellow towards the base, white at base; surface smooth, pruinose, covered with small white flakes, solid or slightly fistulose. ***Context*** up to 1–2 mm thick, greenish white in the pileus, reddish white in the stipe, immutable. ***Odor*** and ***taste*** indistinct.

***Basidiospores*** strongly dimorphic: *macrospores* (13.5–)15.7–*17.5*–19.4(–21.0) × (8.0–)9.1–*10.1*–11.0(–12.0) µm, [95/6/3], *Q* = 1.60–*1.75*–1.90, *V* = 689–*948*–1206 µm^3^, oblong, rarely ellipsoid, sometimes with a subplane adaxial side, inamyloid, thin- to slightly thick-walled (wall up to 1 µm), hyaline, mostly with refractive guttulate content, occasionally with a large inner guttula, without a suprahilar depression, with mostly obtuse apex, and distinct hilar appendix; *microspores* (5.0–)5.9–*6.5*–7.2(–8.0) × (3.0–)3.5–*3.96*–4.4(–5.0) µm [120/6/3], *Q* = 1.50–*1.65*–1.81, *V* = 39.0–*54.9*–70.9 µm^3^_,_ ellipsoid to oblong, inamyloid, thin- to slightly thick-walled [wall up to 0.5 (–0.8) µm thick], hyaline, with one or more guttules and with evident hilar appendix. ***Basidia*** clearly dimorphic: *macrobasidia* (30.0–)37.0–*43.8*–50.6(–57.5) × (12.0–)14.1–*16.3*–18.4(–21.0) µm [41/6/3], clavate, ventricose, often with more or less long peduncle, with large medallion clamp at base, tetrasporic to bisporic, sterigmata up to 8 μm long, thin- to thick-walled (wall up to 1 µm thick), hyaline, guttulate; *microbasidia* (17.0–)20.8–*24.4*–28.0(–31.5) × (5.0–)6.0–6.8–7.6(–8.5) μm [77/6/3], *Q* = 3.0–*3.6*–4.3, *V* = 428–*607*–786 µm^3^, narrowly clavate to clavate, mostly with more or less long peduncle, with medallion clamp at base, tetrasporic to bisporic, sterigmata up to 9(–10) μm long, thin-walled to slightly thick-walled (wall up to 0.5 µm thick) apically, hyaline and often internally guttulate. ***Pleurocystidia*** absent. ***Lamellar edge*** shows pseudocystidia (sterile elements that originate from the hymenophoral trama hyphae) and cheilocystidia. ***Pseudocystidia*** 30–120 × 8–15 μm, subcylindrical, lageniform, subfusiform, sometimes reclined, straight or sinuous, sometimes septate, thin-walled, hyaline, often with refractive guttulate deposits, with rounded to subacute apex. ***Cheilocystidia*** abundant, mostly localized near the stipe, broadly clavate to subcylindrical, 20–40 × 8–15 µm, with a more or less long peduncle, hyaline, without inner guttulae. ***Hymenophoral trama*** regular to subregular; a mixture of short, clavate, swollen, fusiform, 20–50 µm wide hyphae and long subcylindrical, often > 500 µm × 5–20(–30) μm hyphae, often tapered towards the septa; thin- to thick-walled (to 1.5 µm thick), faintly yellowish colored, with a hyaline to pale yellowish cytoplasmic pigment; rare thromboplerous hyphae, 2–5 µm wide, sinuous and with yellowish contents. ***Pileipellis*** as a subregular cutis, consisting of a) mostly fusiform parallel (repent) hyphae, over 200 µm long, 10–20 µm wide, some with an adscending terminal element, with yellowish-ochre cytoplasmic contents; b) thin hyphae with an irregular pattern, intertwined, exceeding 200 µm long, 3–5 µm wide, thin-walled, hyaline or slightly greenish under optical microscopy. Discontinuously, there are also tufts of emerging pileocystidioid hyphae with a morphology similar to that of pseudocystidia (see picture ANGE1940). Occasional thromboplerous hyphae with yellowish contents. ***Subcutis*** subregular to irregular, made up of short fusiform, subvesicular elements, and fusiform hyphae even over 200 µm long and 20–40(–50) μm wide, thin-walled. ***Stipitipellis*** a cutis composed of parallel cylindrical hyphae, 3–10 μm wide, thin to slightly thick-walled (to 1.0 µm thick), with a yellowish cytoplasmic pigment, producing, especially at the stipe apex, copious and characteristic tufts of **caulocystidia** 20–60 × 6–20 µm which have a morphology similar to those present on the lamellar edge near the stipe. There are also thin hyphae with an irregular pattern, intertwined, exceeding 200 µm long, 3–5 µm wide, thin-walled, hyaline or slightly greenish, similar to those found in the pileipellis. ***Stipititrama*** regular, consisting of parallel hyphae even over 200 µm long, 5–15(–20) μm wide, thin-walled, mostly hyaline. ***Clamp-connections*** present throughout.

#### Habit, habitat, occurrence, and distribution.

Growing solitary on litter in mountain forests with conifers (*Pinus occidentalis* Sw.). Quite common.

#### Additional specimens examined.

Dominican Republic, La Vega, Jarabacoa 19.188667, –70.592655, 7 December 2016, C. Angelini (ANGE888); *ibidem*, 12 December 2023 (ANGE1940).

#### Notes.

This species is characterized by its medium-sized basidiomata, a radially fibrillose pileus, a long stipe, yellowish-reddish-green tinges on the pileus, lamellae, and stipe, and the presence of macro- and microbasidiospores. The three nrITS sequences of *H. jarabacoensis* clustered in a highly supported clade (SH-aLRT = 84, UFB = 100, BPP = 0.98). In the phylogeny, the /*H. jarabacoensis* clade is sister to the sequence of *H. chloochlora* from Puerto Rico (SAC-PR-9899, GenBank KF381522). The nrITS sequence from the holotype of *H. jarabacoensis* revealed 95% sequence identity (578 of 609 base pairs) and a gap occurrence of 3% (19 of 609 base pairs) when compared with the nrITS sequence of *H. chloochlora* from Puerto Rico (GenBank KF381522). Both are placed within a more inclusive supported clade (SH-aLRT = 92, UFB = 98, BPP = 0.99) that includes *H. appalachianensis* (TENN:61606) and four sequences from Belize deposited in GenBank as *H. aff. chloochlora* (CFMR BZ-3042, EU435147, *Hygrocybe smaragdus* Lodge, T.J. Baroni & Aime, nom. prov., ined.), *H. aff. prieta* (CFMR BZ-3099, GenBank KF291168, *Hygrocybe elongatipes* Lodge, T.J. Baroni & Aime, nom. prov., ined.; BRH; CFMR), and *H. aff. neofirma* (CFMR BZ-1926, KF291166; BRH; PUL).

Among the species of *Hygrocybe* with green coloration, *H. jarabacoensis* differs from *H. chloochlora* Pegler & Fiard from the Caribbean and *H. aff. chloochlora* from Belize in lacking a membranous marginal fringe on the pileus; it also lacks a perforated pileus, whereas *H. chloochlora* usually has a pileus perforated at the center (Pegler and Fiard 1978; Pegler 1983; Lodge and Pegler 1990; Lodge, pers. obs.). In addition, the macrospores of *H. jarabacoensis* (14–21 × 8–11 µm) are larger than those of *H. aff. chloochlora* from Belize (10.7–15.2 × 6.4–8.4 µm). Among *Hygrocybe* species in section *Pseudofirmae* from the Caribbean Basin with purple or dusky tints on the pileus, *H. jarabacoensis* shares the presence of waxy, furfuraceous scales on the stipe with *H. prieta* Lodge & Pegler but differs in lacking a perforated pileus with a sterile fringe and in having larger macrospores (14–21 × 8–11 vs. 10–17 × 7–9 µm) (Lodge and Pegler 1990).

### Hygrocybe parabolica

Taxon classificationFungiAgaricalesHygrophoraceae

Angelini, Dovana, Lodge, M. Marchetti & Vizzini sp. nov.

84545603-4B25-565D-AC2B-73293C27131A

864519

Fig. 7

#### Etymology.

The specific epithet refers to the parabolic shape of its pileus.

**Figure 7.**
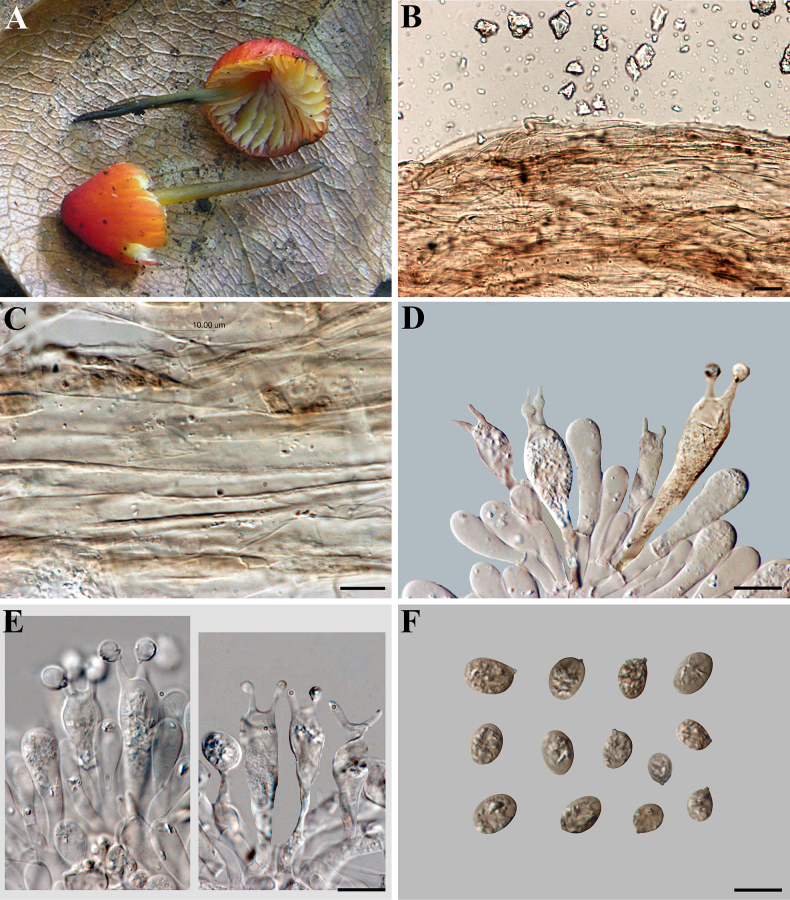
*Hygrocybe parabolica* (JBSD 141877, holotype). **A** Fresh basidiomata; **B** pileipellis; **C** elements of the hymenophoral trama; **D** hymenium (macrobasidia, microbasidia, and basidiola); **E** macrobasidia (left) and microbasidia (right); **F** basidiospores (macrospores and microspores at the bottom right). **B–F** in ammoniacal Congo red. Photos by C. Angelini (**A**) and M. Marchetti (**B–F**). Scale bars: 10 μm.

#### Diagnosis.

Differing from *H. singeri* in the less viscid (subviscid) basidioma, the slow and faint blackening of the entire basidioma upon aging, which, on the stipe, is restricted solely to the base, pale yellow lamellae, wider basidiospores, 2(–4)-spored basidia, presence of microspores, and divergence in ribosomal sequences.

#### Types.

Dominican Republic, Puerto Plata, Sosúa, loc. Cemetery 19.746151, –70.540367, 23 December 2016, C. Angelini, JBSD 141877 Holotype (ANGE821 Isotype). GenBank: ITS = PZ148587.

#### Description.

***Pileus*** up to 15 mm in diam., parabolic or broadly parabolic, usually not fully expanded, with a rounded umbo, margin even, not lobed; surface smooth, viscid when fresh then moist or dry, orange-red, light pellucid orange and striate at margin, blackening slowly with age. ***Lamellae*** adnexed, sometimes slightly emarginate, ventricose, up to 3 mm broad, intercalated with lamellulae, pale yellow, thick, and distant, with irregular and concolorous edge. ***Stipe*** 25–30 × 2–3 mm, cylindrical, hollow, olive yellow; surface subviscid when collected, possibly more viscid based on the deeply gelatinized stipiticutis, blackening when handled or with age mostly at the base. ***Context*** very thin in the pileus. ***Odor*** and **taste** indistinct.

***Basidiospores*** mostly as *macrospores* (8.0–)8.8–*9.7*–10.5(–11.5) × (5.5–)6.5–*7.2*–7.8(–8.5) µm [107/1/1], *Q* = 1.23–*1.35*–1.48, *V* = 199–*264*–329 µm^3^ (from bisporic basidia), broadly ellipsoid to ellipsoid with rounded apex (rarely tapering towards the apex) in frontal view, sometimes with a sub-flat adaxial in lateral view, without median constriction, hyaline, smooth, thin-walled, with broad and obtuse hilar appendix, guttulate; *microspores* rare, (6.5–)7.1–*7.4*–7.7(–8.0) × (5.5–)6.1–*6.5*–6.9(–7.5) µm [11/1/1], *Q* = 1.08–*1.14*–1.21, *V* = 142–*165*–189 µm^3^, subglobose to broadly ellipsoid, with prominant hilar appendix, with uniform or guttulate contents. ***Basidia*** mostly as *macrobasidia* (29.5–)33.2–*37.1*–40.9(–45.0) × (8.0–)8.8–*9.7*–10.7(–11.5) µm [27/1/1], *Q* = 3.4–*3.8*–4.3, *V* = 1456–*1866*–2277 µm^3^, clavate to cylindrical-clavate, sometimes with a long pedicel (up to 15 (–20) µm long) tapering downwards, bisporic (abundant) or tetrasporic (rare), monosporic (very rare), often with guttulate content, sterigmata up to 8(–10) µm long; *microbasidia* occasional (very rare) (21.5–)24.1–*26.7*–29.3(–32.0) × (6.5–)7.35–*8.32*–9.3(–10.5) µm [19/1/1], *Q* = 2.9–*3.2*–3.5, *V* = 696–*994*–1292 µm^3^, cylindrical-clavate, hyaline, guttulate. ***Lamellar edge*** fertile. ***Cheilocystidia*** and ***Pleurocystidia*** not observed. ***Hymenophoral trama*** regular, hyphae parallel, subcylindrical, mostly longer than 200 µm, some up to 1000 µm long, 5–20 µm wide, hyaline or faintly yellow, with the wall often up to 2.0 µm thick and then yellowish in color. ***Pileipellis*** an ixocutis with radial, repent cylindrical hyphae up to 80 µm long, 3–7 µm wide, showing a brownish, solid or dissolved cytoplasmic pigment and an epiparietal pigment as darker colored plaques. ***Subcutis*** regular, consisting of subparallel, subcylindrical hyphae, often over 200 µm long mixed with shorter elements, 5–14 µm wide, hyaline to pale yellowish. ***Stipitipellis*** as a strongly gelatinized ixocutis of parallel subcylindrical hyphae, up to 100 µm long, 3–6 μm wide, thin- to slightly thick-walled (wall up to 1.0 μm thick), with pale to brownish or reddish-brown cytoplasmic pigment; ***caulocystidia*** absent. ***Stipititrama*** regular, made up of parallel, subcylindrical hyphae, often over 200 µm long mixed with shorter elements, 5–15(–18) µm wide, hyaline to faintly brownish. ***Clamp-connections*** observed on all hyphae especially at the base of the basidia.

#### Habit, habitat, occurrence, and distribution.

Growing gregariously on burned ground in broad-leaved forest a few kilometers from the sea. Uncommon and localized.

#### Notes.

*Hygrocybe parabolica* is characterized by its small-sized basidiomata, a parabolic red-orange pileus with a non-lobed margin, a subviscid basidioma, and slow blackening, mostly localized to the stipe. Microscopically, it is characterized by predominantly bisporic basidia and a lack of clear distinction between microspore and macrospore measurements. *Hygrocybe parabolica* belongs to the /conica clade and is the sister species of *H. singeri* (A.H. Sm. & Hesler) Singer, sharing 93% identity (550/593) with 12/593 gaps (2%). *Hygrocybe singeri* differs from *H. parabolica* in being so viscid that it was difficult to hold and in blackening entirely vs. mostly in the stipe; in having initially light-orange vs. pale yellow lamellae; in having narrower spores (5–6 vs. 6–7 µm) that are slightly bean-shaped in side view; in having exclusively four-spored basidia; and in lacking microspores (Smith and Hesler 1954). *H. singeri var. albifolia* (Hesler & A.H. Sm.) Singer resembles *H. parabolica* in having bisterigmate basidia and broader spores (× 6–7.5 µm), but it differs in having a larger pileus (20–50 vs. up to 15 mm) and paler lamellae (white vs. pale yellow). The type variety of *H. singeri* is represented by a single sequence in GenBank from the isotype, whereas *H. singeri var. albifolia* is known only from the type and is entirely blackened after being pressed; therefore, neither is well circumscribed.

### Hygrocybe perdomoi

Taxon classificationFungiAgaricalesHygrophoraceae

Angelini, Dovana, Lodge, M. Marchetti & Vizzini sp. nov.

93232BE2-CBDF-5B41-B780-9652FF32116B

864520

Figs 8, 9, 10

#### Etymology.

Dedicated to the Dominican mycologist Omar Paino Perdomo.

**Figure 8.**
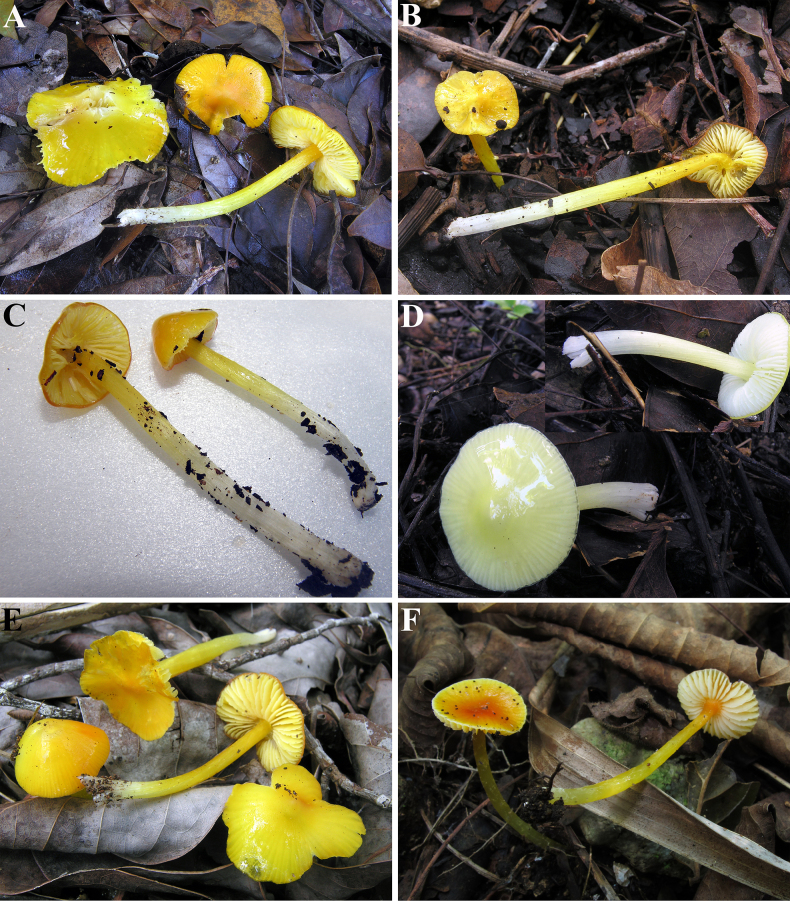
*Hygrocybe perdomoi*, fresh basidiomata. **A**JBSD 141873; **B** ANGE815; **C** ANGE816; **D** ANGE818; **E** 15 December 2019; **F** 21 January 2010. Photos by C. Angelini.

**Figure 9.**
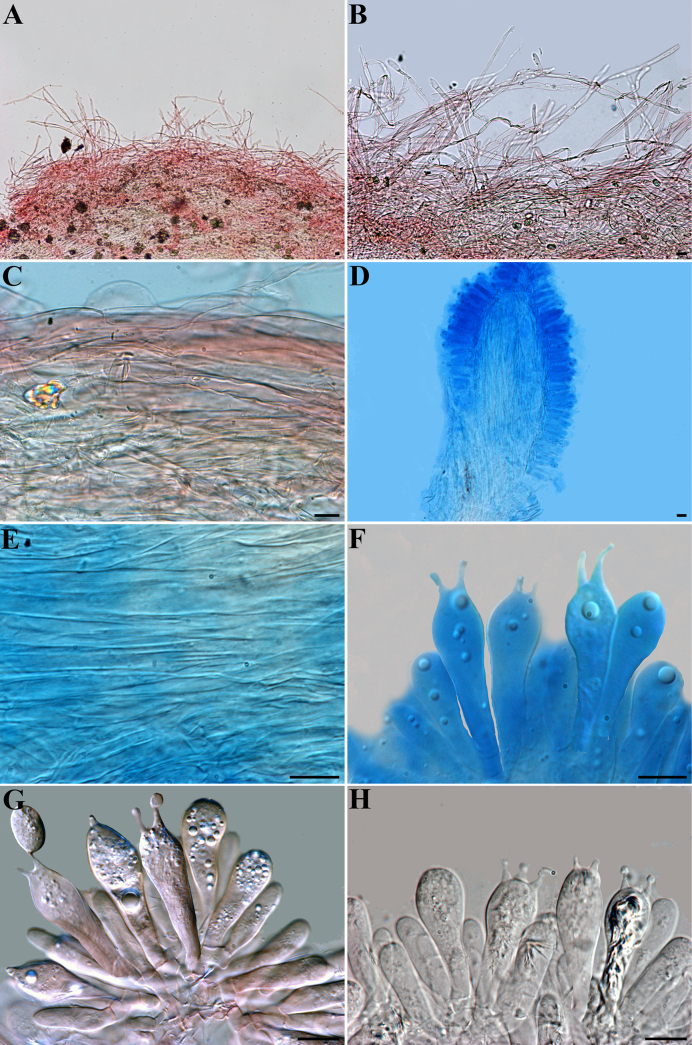
*Hygrocybe perdomoi*, microscopic features. **A–B** Pileipellis (ANGE816, JBSD 141873, holotype); **C** subcutis (JBSD 141873, holotype); **D** microscopic section of a lamella (JBSD 141873, holotype); **E** elements of the hymenophoral trama (JBSD 141873, holotype); **F–H** basidia and basidiola (JBSD 141873, holotype). **A–C**, **G–H** in ammoniacal Congo red; **D–F** in cotton blue. Photos by M. Marchetti. Scale bars: 10 μm.

**Figure 10.**
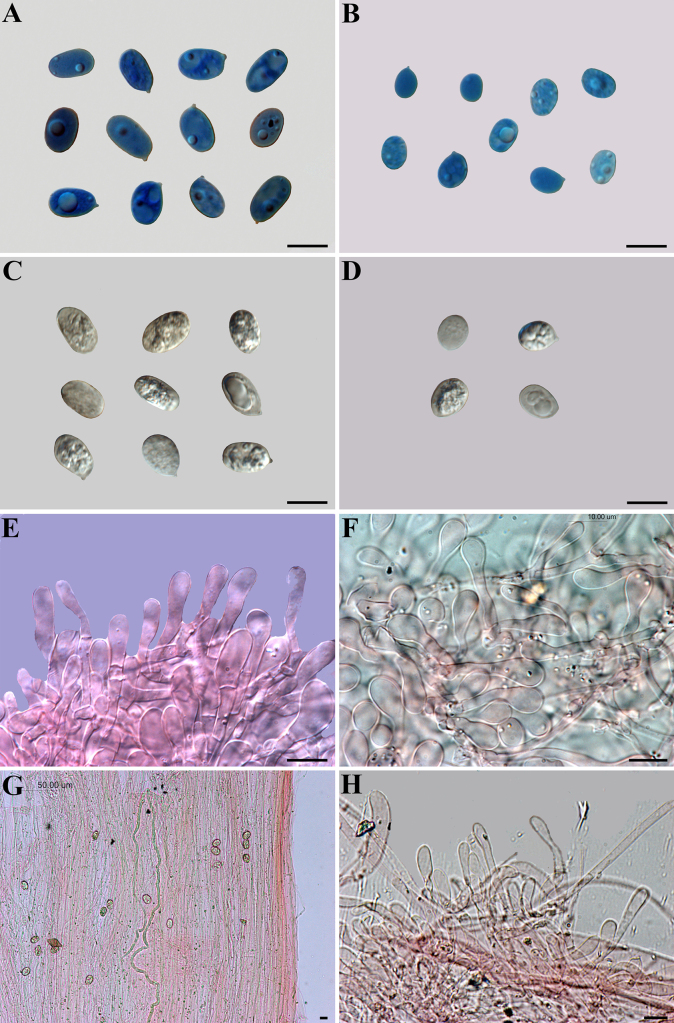
*Hygrocybe perdomoi*, microscopic features. **A** Macrospores (JBSD 141873, holotype); **B** microspores (JBSD 141873, holotype); **C** macrospores (ANGE815); **D** microspores (ANGE815); **E** pseudocystidia and basidiola (JBSD 141873, holotype); **F** pseudocystidia (ANGE816); **G** stipititrama with thromboplerous hyphae (JBSD 141873, holotype); **H** stipitipellis with caulocystidia-like hyphae (JBSD 141873, holotype). **A–B** in cotton blue; **C–D**, **F–H** in ammoniacal Congo red; **E** in phloxine. Photos by M. Marchetti. Scale bars: 10 μm.

#### Diagnosis.

Differing from *H. acutoconica* in smaller slimy-viscid basidioma, not constricted broadly ellipsoid, ellipsoid or ellipsoid-ovoid basidiospores, and divergence in ribosomal sequences.

#### Types.

Dominican Republic, Puerto Plata, Sosúa, loc. Cemetery 19.746151, –70.540367, 11 December 2016, C. Angelini, JBSD 141873 Holotype (ANGE813 Isotype). GenBank: ITS = PZ148589, LSU = PZ145852.

#### Description.

***Pileus*** 15–42 mm diam., conical at first, later expanded to applanate, with an umbo, sometimes cuspidate (e.g., Mushroom Observer 501267, GenBank PP383936, 99.13% similar) margin not lobed; surface smooth, viscid and shiny, with striate margin up to more than half the radius, surface pale Straw Color, light lemon yellow, golden yellow, to orange yellow in the Dominican Republic, orange yellow, orange or scarlet red in Florida (iNaturalist 195601389; GenBank PP850544, 99.27% similar, Mushroom Observer 501267). ***Lamellae*** narrowly adnexed, ventricose, up to 7 mm broad, intercalated with 1–2 lengths of lamellulae, pale yellow to Cream color or white, rather thin, distant, with pale, irregular eroded edge. ***Stipe*** 40–70 × 3–5 mm, cylindrical, straight or curved, usually yellow and white; surface viscid, fibrillose-ribbed and twisted, covered with scattered white floccules. ***Context*** up to 2 mm thick, yellowish orange in the pileus, yellowish white in the stipe. ***Odor*** and ***taste*** indistinct.

***Basidiospores*** (8.0–)9.6–*10.9*–12.3(–13.5) × (5.5–)6.3–*7.3*–8.3(–9.5) µm [230/6/3], *Q* = 1.35–*1.51*–1.67, *V* = 210–318–426 µm^3^, broadly ellipsoid, ellipsoid, ellipsoid-ovoid, oblong, without ventral constriction, smooth, hyaline, thin-walled, guttulate, with broad and obtuse hilar appendix. In some specimens occasional presence (copious to scarce) of less voluminous, ellipsoid basidiospores (6.4–)7.3–*8.2*–9.0(–9.9) × (4.7–)5.4–*6.1*–6.7(–7.4) µm [55/4/2], *Q* = 1.25–*1.35*–1.45, *V* = 110–*162*–210 µm^3^_._***Basidia*** (31.2–)34.8–*38.4*–42.1(–45.7) × (10.3–)11.0–*11.7*–12.5(–12.5) µm [32/2/1], *Q* = 3.0–*3.3*–3.6, *V* = 2323–*2797*–3271 µm^3^_,_ clavate, cylindrical-clavate, with tapering base, in some basidiomata predominantly tetrasporic, in others bisporic, monosporic ones are also present, often guttulate with large or small guttulae, sterigmata up to 8 µm long. ***Basidiola*** subcylindrical to broadly clavate, 30–35 × 10–12 µm. ***Lamellar edge*** fertile. ***Cheilocystidia*** and ***Pleurocystidia*** absent***. Pseudocystidia***, occasional (rare), mostly on the lamellar edge near the attachment point of the stipe, arising as a final extension of the hymenophoral trama hyphae, 15–40 × 7–12 µm, clavate, swollen, subcylindrical. ***Hymenophoral trama*** regular, consisting of parallel long tubular elements, usually 120–350 × 3–8 (–10) µm, sometimes even longer, with an enlarged end; present also hyphae with refractive cytoplasmic content. ***Pileipellis*** as an irregular ixotrichoderm (scarce gelled matrix), with emerging, erect or semi-erect hyphae, loose, sub-intertwined, multi-articulate (pluriseptate), simple or bifurcated, exceeding 150–200 µm and 3–7 µm broad, terminal element usually swollen, sometimes clavate. ***Subcutis*** made up of subcylindrical to fusiform hyphae, exceeding 200 µm and 8–15 (–20) µm broad, with hyaline to slightly yellowish content; thromboplerous hyphae abundant, almost straight to wavy, 5–12 µm broad, with greenish content; copious presence of refractive crystalline deposits. ***Stipitipellis*** as an ixocutis consisting of parallel to slightly intertwined, 3–8 μm broad hyphae, thin- to slightly thick-walled (wall up to 0.5 (0.8) µm thick), with a pale yellowish cytoplasmic pigment; in the apical portion of the stipe there are tufts of club-shaped caulocystidia-like hyphae, 40–60 × 5–10 µm, hyaline, thin-walled. ***Stipititrama*** formed by parallel subcylindrical hyphae, even longer than 200 µm, 8–15 (–20) µm broad, with small or large refractive crystalline deposits. ***Clamp-connections*** present everywhere.

#### Habit, habitat, occurrence, and distribution.

Growing in small groups on litter in broad-leaved forests a few kilometers from the sea. Common. According to the phylogeny, known from the Dominican Republic and Florida in southeastern USA.

#### Additional specimens examined.

Dominican Republic, Puerto Plata, Sosúa, loc. Cemetery 19.746151, –70.540367, 22 December 2016, C. Angelini (ANGE816); *ibidem*, 22 December 2016, C. Angelini (ANGE818); *ibidem*, 3 December 2016, C. Angelini (ANGE815); *ibidem*, 21 January 2010, C. Angelini (dried collection lost); *ibidem*, 15 December 2019, C. Angelini (dried collection lost).

#### Additional collections matched using a GenBank BLAST search.

USA, Florida, Alachua County, Gainseville, Sweetwater Preserve, off of entrance on 16th street, lat. 29.629, long. –82.32, 6 August 2017, B. Kaminsky & G. LaPierre, GenBank MH211824, FLAS-F-61222; Miami-Dade County, Everglades National Park, lat. 25.615, long. –80.584, 19 June 2020, A.R. Franck, iNaturalist 50221983, GenBank OP580224; *ibid*., 19 June 2020, A.R. Franck, ARF-4859, iNaturalist 195601387, GenBank OP580224; FLAS-F-70002; Palm Beach County, Jupiter Inlet Lighthouse, lat. 26.95, long. –80.08, 2 January 2024, S. Otsuni, iNaturalist 195601389, GenBank PP850544; *ibid*, iNaturalist 195601387, GenBank PP791118; Volusia County, DeLand, Lake Beresford Park, lat. 29.0, long. –81.344, 9 September 2022, S. Otsuni, iNaturalist 134321825; Mushroom Observer 501267, GenBank PP383936.

#### Notes.

*Hygrocybe perdomoi* is characterized by its small, entirely viscid basidiomata; a pileus with a striate margin, colored vivid yellow to orange in collections from the Dominican Republic or orange to scarlet red in collections from Florida; and a viscid, fibrillose-ribbed surface of the stipe. *Hygrocybe pseudohypohaemacta* differs in having entirely glutinous basidiomata, white or rarely pinkish-orange-red vs. cream to pale yellow lamellae, and basidiospores that are subglobose to broadly ellipsoid vs. ellipsoid, broadly ellipsoid, or ellipsoid-ovoid. The four sequences of *H. perdomoi* from the Dominican Republic, together with five sequences retrieved from GenBank originating from Florida in the southeastern USA, formed a well-supported clade (SH-aLRT = 94, UFB = 98, BPP = 1) within the *Hygrocybe* /acutoconica clade. The /acutoconica clade itself is a polytomous, strongly supported group (SH-aLRT = 100, UFB = 100, BPP = 1), containing six additional sequences representing *H. acutoconica var. pallidocarnea*, *H. acutoconica var. persistens*, H. acutoconica f. japonica, and two distinct terminal taxa identified as *H. konradii var. konradii*. Within the /acutoconica clade, only the /*H. perdomoi* subclade includes sequences from both North and Central America; all other sequences originate from specimens collected in Europe or Japan. *Hygrocybe acutoconica* (Bon 1990; Candusso 1997; Boertmann 2010) differs from *H. perdomoi* in having more robust basidiomata (pileus up to 70 mm diam., stipe up to 10 mm thick), with a moist or slightly viscid pileus vs. slimy viscid, a dry vs. slimy-viscid stipe surface, and spores that are cylindrical to oblong (mean *Q* = 1.8–2.2) and sometimes constricted vs. mostly broadly ellipsoid, ellipsoid, or ellipsoid-ovoid (mean *Q* = 1.46–1.56) and not constricted.

### Hygrocybe pseudohypohaemacta

Taxon classificationFungiAgaricalesHygrophoraceae

Angelini, Dovana, Lodge, M. Marchetti & Vizzini sp. nov.

222FD2BE-5AF1-539E-B010-AEDACF013958

864521

Figs 11, 12, 13

#### Etymology.

The specific epithet is derived from the Greek prefix pseudo- (false), referring to its close morphological similarity to *H. hypohaemacta*, with which it may be easily confused.

**Figure 11.**
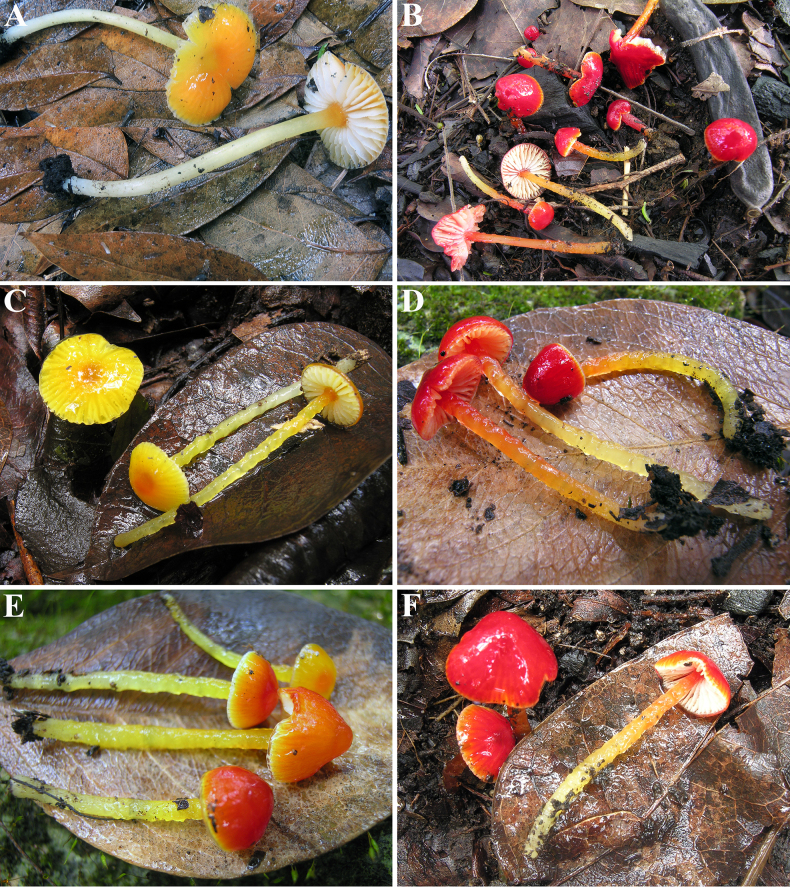
*Hygrocybe* sp. (ANGE436). **A** Fresh basidiomata. *Hygrocybe pseudohypohaemacta*. **B–F** Fresh basidiomata (JBSD 141878, holotype, ANGE822, ANGE823, ANGE824, ANGE825). Photos by C. Angelini.

**Figure 12.**
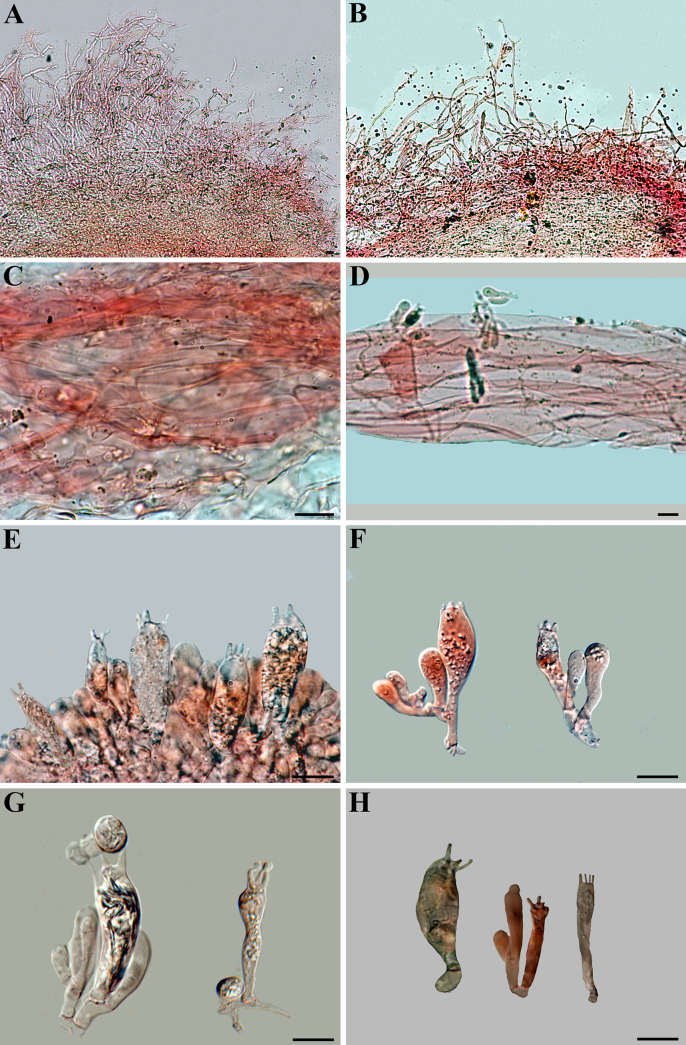
*Hygrocybe pseudohypohaemacta*, microscopic features. **A–B** Pileipellis (JBSD 141878, holotype); **C** subcutis (ANGE822); **D** hymenophoral trama (JBSD 141878, holotype); **E** dimorphic basidia (JBSD 141878, holotype); **F–H** macrobasidia (left) and microbasidia (right) (JBSD 141878, holotype, ANGE823, ANGE822). **A–H** in ammoniacal Congo red. Photos by M. Marchetti. Scale bars: 10 μm.

**Figure 13.**
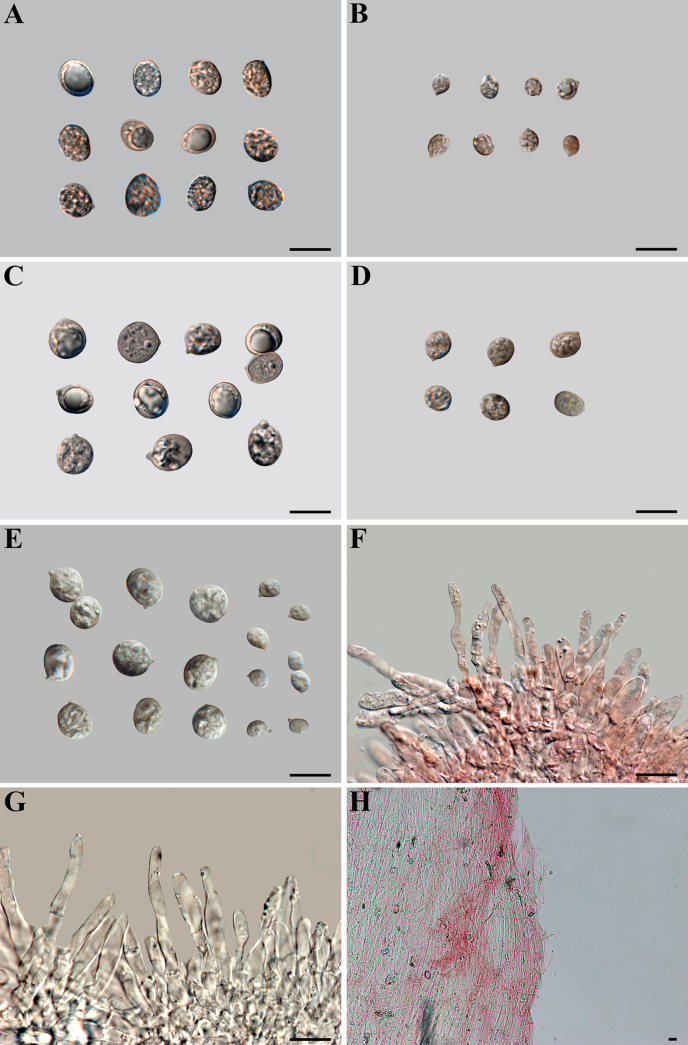
*Hygrocybe pseudohypohaemacta*, microscopic features. **A** Macrospores (JBSD 141878, holotype); **B** microspores (JBSD 141878, holotype); **C** macrospores (ANGE823); **D** microspores (ANGE823); **E** macrospores (left) and microspores (right) (ANGE824); **F–G** pseudocystidia (JBSD 141878, holotype, ANGE825); **H** stipitipellis (JBSD 141878, holotype). **A–H** in ammoniacal Congo red. Photos by M. Marchetti. Scale bars: 10 μm.

#### Diagnosis.

Differing from *H. hypohaemacta* in larger microspores, differently shaped macrospores, absence of hymenial cystidia, geographic range, and divergence in ribosomal sequences.

#### Types.

Dominican Republic, Puerto Plata, Sosúa, loc. Cemetery 19.746151, –70.540367, 23 December 2016, C. Angelini, JBSD 141878 Holotype (ANGE819 Isotype). GenBank: ITS = PZ148603, LSU = PZ145854.

#### Description.

***Pileus*** 15–28 mm diam., at first and for a long time conical or hemispherical, later expanded to applanate, without an umbo and the margin not or very slightly lobed; surface smooth, glutinous and shiny, with translucent-striate margin up to more than half the radius, uniformly colored bright scarlet red or orange, but always with a narrow yellow margin, some yellow with a reddish-orange center and yellow margin. ***Lamellae*** narrowly adnate, just free or sometimes slightly emarginate, ventricose, up to 2 mm broad, intercalated with 2 lengths of lamellulae, white, rarely pale pinkish-orange-red, thick, and distant, with irregular and concolorous edge. ***Stipe*** 30–60 × 1–4 mm, cylindrical, straight or curved, often tapered towards the apex, usually partly golden-yellow, sometimes reddish-yellow or reddish orange; surface covered by a very thick transparent glutinous layer (as in *Roridomyces* Rexer, *Mycenaceae* Overeem). ***Context*** thin, reddish white in the pileus, yellowish white in the stipe. ***Odor*** and ***taste*** indistinct.

***Basidiospores*** dimorphic; *macrospores* (7.5–)8.2–*9.1*–9.9(–11.0) × (6.0–)7.0–*7.8*–8.7(–9.5) µm [246 10/6] *Q* = 1.07–*1.16*–1.26, *V* = 214–*300*–385 µm^3^, subglobose to broadly ellipsoid, inamyloid, thin- to thick-walled (wall up to 0.5 µm thick), hyaline, mostly with guttulate content and with a large central guttula; *microspores* (4.2–)4.8–*5.3*–5.9(–6.4) × (3.2–)3.8–*4.4*–5.0(–5.6) µm [141/10/5], *Q* = 1.09–*1.22*–1.35, *V* = 35–*56*–78 µm^3^, subglobose to broadly ellipsoid, inamyloid, thin- to thick-walled (wall up to XX thick µm), hyaline, with only a large central guttula or multiguttulate. ***Basidia*** dimorphic; *macrobasidia* (23.8–)27.7–*31.3*–35.6(–39.5) × (8.4–)9.5–*10.5*–11.6(–12.6) [33/3/2], *Q* = 2.6–*3.0*–3.4, *V* = 1398–*1860*–2322 μm^3^, clavate, usually squat, with or without peduncle, thin-walled or slightly thick-walled at apex, hyaline, with guttulate content, tetrasporic and bisporic; sterigmata up to 8 μm long; *microbasidia* (15.7–)18.8–*21.9*–25.0(–28.5) × (4.4–)5.2–*6.0*–6.8(–7.6) μm [141/10/5], *Q* = 3.1–*3.7*–4.2, *V* = 286–*429*–573 µm^3^, narrowly clavate to clavate, mostly with a more or less long peduncle, thin-walled or slightly thick-walled at apex, hyaline, with guttulate content, tetrasporic to bisporic, sterigmata up to 7 μm long. The ratio between macrobasidia and microbasidia varies across different collections. ***Pleurocystidia*** and ***cheilocystidia*** absent. **Lamellar edge** fertile with pseudocystidia arising deep in the hymenophoral trama. ***Pseudocystidia*** 15–40(–50) × 4–6.5 μm, abundant, clavate to subcylindrical, usually sinuous, mostly septate, sometimes bifurcated, with rounded to acute apex, sometimes subcapitate, sometimes with crystalline deposits in the apical portion, thin-walled, hyaline, with smooth or minutely guttulous content. ***Hymenophoral trama*** regular to subregular, consisting of parallel hyphae usually > 200 µm long and 5–20 μm wide, thin-walled, hyaline; occasional thromboplerous hyphae with refractive greenish content are present. ***Pileipellis*** as an irregular ixotrichoderm, hyphae erect to semi-erect, parallel to intertwined, 2–5 μm wide, frequently exceeding 200 µm in length and multi-articulate (pluriseptate), hyaline, to pale yellowish, terminal elements sometimes enlarged at apex or clavate. ***Subcutis*** subregular, hyphae frequently exceeding 200 µm in length, 3–15(–30) μm wide, thin-walled, with a pale yellowish cytoplasmic pigment and crystalline deposits. ***Stipitipellis*** a mixed ixocutis-ixotrichodermium with abundant gelatinous matrix, consisting of parallel or slightly intertwined hyphae, some over 200 µm long, scarcely septate, 2–7 μm wide, thin- to slightly thick-walled (wall up to 1.0 (–1.5) μm thick), hyaline or with a pale yellowish cytoplasmic pigment; especially in the apical portion of the stipe, dense tufts of erect, semi-erect interwoven hyphae in gelled material, with a morphology similar to pileal elements. ***Stipititrama*** regular to subregular, gelatinized some over 200 µm long, 5–15(–20) μm wide, scarcely septate, thin-walled, showing a pale yellowish cytoplasmic pigment and crystalline deposits on the outside. ***Clamp-connections*** present throughout.

#### Habit, habitat, occurrence, and distribution.

Growing gregariously, in groups of a few to many specimens, on litter in broad-leaved forests, sometimes near burned ground (charcoal-pit residue), a few kilometers from the sea. Apparently uncommon and localized. Thus far known from the Dominican Republic, Belize, and French Guiana based on molecular data.

#### Additional specimens examined.

BELIZE, Orange Walk District, La Milpa Field Station, Lagunita Trail, lat. 17.84, long. –89.02, 26 October 2002, D.J. Lodge & B. Ortiz-Santana, DJL-BZ-105, CFMR BZ-1903, GenBank U435150; BRH; CFMR. DOMINICAN REPUBLIC, Puerto Plata, Sosúa, loc. Cemetery 19.746151, –70.540367, 25 December 2016, C. Angelini, JBSD 141874 (ANGE823 duplicate) *ibidem*, 25 December 2016, C. Angelini (ANGE822); *ibidem*, 25 December 2016, C. Angelini (ANGE824); *ibidem*, 25 December 2016, C. Angelini (ANGE825).

#### Notes.

*Hygrocybe pseudohypohaemacta* is characterized by its small-sized, entirely slimy basidiomata; a vivid scarlet-red or orange pileus with a yellow, striate margin; and a stipe covered with a thick, transparent, glutinous layer. Pseudocystidia 15–40(–50) × 4–6.5 μm, abundant, clavate to subcylindrical, usually sinuous, mostly septate, sometimes bifurcated, with a rounded to acute apex, sometimes subcapitate, sometimes with crystalline deposits in the apical portion, thin-walled, hyaline, with smooth or minutely guttulate contents. Six sequences of *H. pseudohypohaemacta* generated in this study, together with two sequences from Belize and French Guiana, formed a well-supported clade (SH-aLRT = 100, UFB = 100, BPP = 1) closely related to *H. variecolor*. *Hygrocybe pseudohypohaemacta*, *H. variecolor*, and other accessions identified as *H. aff./cf. hypohaemacta* and *Hygrocybe* sp. clustered within a well-supported section *Velosae* clade (SH-aLRT = 100, UFB = 100, BPP = 1), together with *H. hypohaemacta*, *H. snigdha* K.P.D. Latha & P. Haridev and *H. pellucida* K.P.D. Latha & Haridev.

*H. pseudohypohaemacta* is morphologically close to *H. hypohaemacta*, but it is well separated in the phylogeny. The nrLSU sequence of *H. hypohaemacta* from Malaysia (KUM61079, GenBank KF381551) showed 93% identity (492/529) with 8 gaps out of 529 (1%) compared with *H. pseudohypohaemacta* (ANGE819) and a patristic distance of 0.34 in the phylogenetic analysis. Based on the original description, the similar *H. hypohaemacta* differs in having smaller microspores [4–5 × 3–3.5 vs. mostly (4.8–)5.3–5.9 × 3.8–5.0 µm], differently shaped macrospores (ovoid to broadly ellipsoid vs. globose, subglobose to broadly ellipsoid), cheilocystidia and caulocystidia at the extreme apex of the stipe, a distribution in Malaysia (Singapore, Corner 1936), and a different nrLSU sequence. *Hygrocybe hypohaemacta* was also reported from Venezuela and Martinique (Pegler 1983), Puerto Rico (Lodge and Pegler 1990), and Panama (Lodge and Ovrebo 2008) based on morphological data.

The nrITS and nrLSU sequences from the Belize collection named *H. hypohaemacta*DJL-BZ-105 (Lodge and Ovrebo 2008) were excluded from the phylogenetic analysis. These sequences showed high BLAST similarity to *Termitomyces* species, indicating that they represent contaminants.

The nrITS sequence of *Hygrocybe* sp. (ANGE436, Fig. 11A) showed 94.41% identity (474/501), with 19 gaps out of 501 (3.7%), compared with *H. pseudohypohaemacta* (JBSD 141878, holotype, ANGE819), and a patristic distance of 0.0093 in the phylogenetic analysis of nrITS and nrLSU. Although the two taxa are morphologically overlapping (data not included), the observed genetic distance and percentage of gaps warrant caution. Therefore, further collections and additional sequences are needed to clarify its taxonomic status and determine whether this taxon represents a distinct species or falls within the variability of *H. pseudohypohaemacta*.

Comparison of the sequences of *H. variecolor* (JBSD 141875) with those of *H. pseudohypohaemacta* (CFMR BZ-1903, GenBank EU435150) revealed 91% identity (117/129) for the nrITS1 region, with 3/129 gaps (2%); 84% identity (132/157) for the ITS2 region, with 15/157 gaps (9%); and 96% identity (804/840) for the nrLSU region, with 13/840 gaps (1%). The data reveal a clear pattern of sequence divergence, with the highest identity in the more conserved nrLSU region (96%), followed by nrITS1 (91%), and the lowest identity in the ITS2 region (84%). Conversely, the percentages of gaps show an inverse trend, with ITS2 exhibiting the highest proportion of gaps (9%), followed by nrITS1 (2%), and nrLSU exhibiting the lowest proportion (1%). This pattern aligns with the generally accepted understanding of the evolutionary rates of these regions, in which the ITS region, particularly the ITS2 subregion in this case, tends to evolve more rapidly than the nrLSU gene. Considering the typical thresholds used in fungal taxonomy, the ITS data, with 91% identity in nrITS1 and 84% identity in ITS2, strongly suggest that *H. variecolor* and *H. pseudohypohaemacta* should be considered distinct species. These identity values fall below the commonly cited 97% threshold for the entire ITS region (Nilsson et al. 2008) and significantly below the higher thresholds suggested for certain genera. The substantial number of gaps, especially in ITS2, further supports this conclusion. Although the nrLSU region shows a higher identity of 96%, which is expected given its more conserved nature, it remains below the stringent 99.8% threshold proposed for species delimitation in some studies of filamentous fungi (Vu et al. 2019).

### Hygrocybe variecolor

Taxon classificationFungiAgaricalesHygrophoraceae

Angelini, Dovana, Lodge, M. Marchetti& Vizzini sp. nov.

AF95F79A-83BD-5775-B78D-CEA5CACF94A4

864523

Fig. 14

#### Etymology.

The specific epithet *variecolor* refers to the pronounced color variability observed both between different specimens and within the same basidioma.

**Figure 14.**
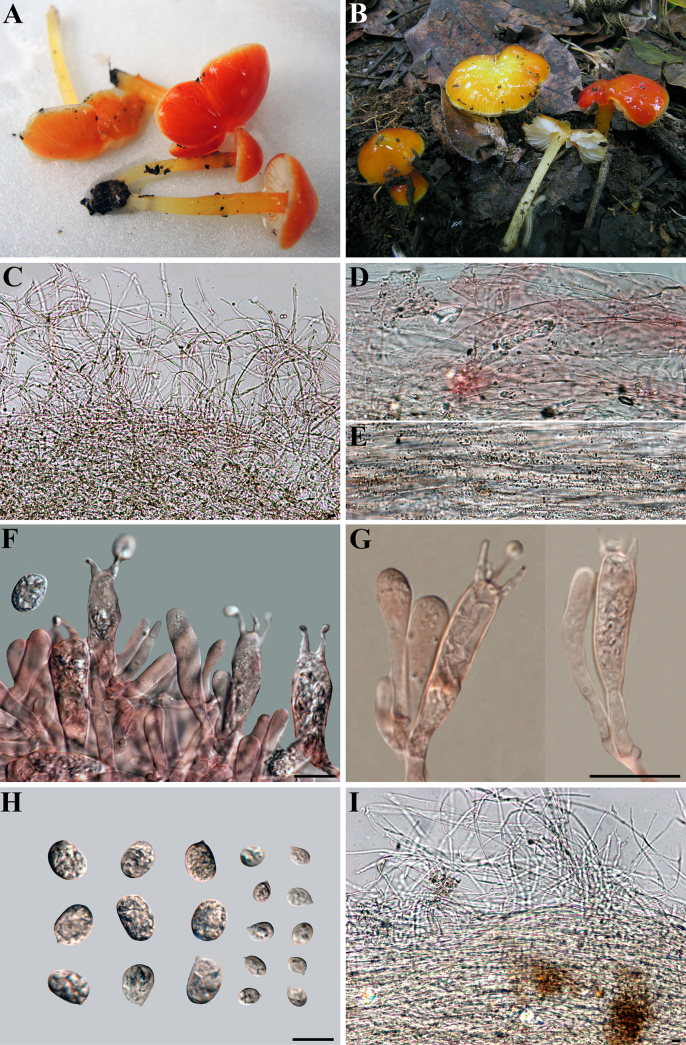
*Hygrocybe variecolor* (JBSD 141875, holotype). **A–B** Fresh basidiomata; **C** pileipellis; **D** subcutis; **E** hyphae of the stipititrama with minute extracellular crystals; **F** macrobasidia and basidiola; **G** microbasidia and basidiola; **H** basidiospores (macrospores, left; microspores, right); **I** stipitipellis. **C–I** in ammoniacal Congo red. Photos by C. Angelini (**A–B**) and M. Marchetti (**C–I**). Scale bars: 10 μm.

#### Diagnosis.

Differing from *H. pseudohypohaemacta* in habitat, larger macro- and microspores with different Q, the rare presence of pseudocystidia, and divergence in ribosomal sequences.

#### Types.

Dominican Republic, Puerto Plata, Sosúa, loc. Puerto Chiquito 19.752653, –70.530561, 23 November 2016, C. Angelini, JBSD 141875 Holotype (ANGE741 Isotype). GenBank: ITS1 = PZ148612, ITS2 = PZ221056, LSU = PZ145856.

#### Description.

***Pileus*** 15–28 mm diam., at first and for a long time conical or hemispherical, later expanded to applanate, without umbo and the margin not or very slightly lobed; surface smooth, glutinous and shiny, with translucent-striate margin up to more than half the radius, uniformly colored bright scarlet red, orange or yellow-orange but always with a narrow yellow margin. ***Lamellae*** narrowly adnate, sometimes slightly emarginate, ventricose, up to 2 mm broad, intercalated with 1 length lamellulae, white, sometimes pale pink-orange-red, thick, and distant, with irregular and concolorous edge. ***Stipe*** 30–60 × 2–4 mm, cylindrical and curved, tapered towards the apex, usually golden-yellow, sometimes reddish-yellow, reddish orange; surface covered by a very thick transparent glutinous layer. ***Context*** very thin, reddish white in the pileus, yellowish white in the stipe. ***Odour*** and ***taste*** indistinct.

***Basidiospores*** dimorphic; *macrospores* (9.0–)9.8–*10.7*–11.5(–12.5) × (6.5–)7.5–8.2–8.9(–10.0) µm [70/2/1], *Q* = 1.20–*1.30*–1.40, *V* = 298–*383*–469 µm^3^, broadly ellipsoid to ellipsoid, rarely subglobose, with the adaxial side sometimes sub-flat, inamyloid, thin- to thick-walled (wall up to 1 µm thick), hyaline, mostly with guttulate content and with a large central guttula; *microspores* (5.0–)5.7–*6.2*–6.6(–7.0) × (4.0–)4.3–*4.8*–5.3(–6.0) µm [53/2/1], Q = 1.13–*1.25*–1.36, V = 62–*81*–100 µm^3^, subglobose to ellipsoid, inamyloid, thin- to thick-walled [wall up to 0.5 (–0.8) µm thick]; hyaline, usually with a large central guttula or with multiple guttulae. ***Basidia*** dimorphic; *macrobasidia* (27.0–)32.1–*37.2*–42.3(–47.5) × (8.5–)9.9–*11.1*–12.3(–13.5) µm [20/2/1], *Q* = 2.87–*3.38*–3.89, *V* = 1724–*2447*–3170 µm^3^, clavate, cylindrical-clavate, often pedunculated, thin-walled or slightly thick-walled (up to 1.0 µm thick) towards the apex, hyaline, usually with guttulate content; tetrasporic and bisporic, sterigmata up to 8 μm long; *microbasidia* (16.0–)19.6–*23.2*–26.7(–30.5) × (4.0–)5.0–*5.7*–6.4(–7.0) μm, *Q* = 3.7–*4.1*–4.4, *V* = 258–*410*–562 µm^3^, narrowly clavate to clavate, mostly with a more or less long (up to 5 (–8) µm) peduncle, thin-walled, hyaline with smooth or guttulate content, tetrasporicc and bisporic, sterigmata up to 8 μm long. ***Pleurocystidia*** and ***cheilocystidia*** absent. **Lamellar edge** fertile, with rare pseudocystidia originate from the hymenophoral trama. ***Pseudocystidia*** 15–40 × 4–6.5 μm, rare, scattered, clavate, subcylindrical, ventricose, with rounded to acute apex, thin-walled, hyaline, with smooth or minutely guttulous content. ***Hymenophoral trama*** regular to subregular; some exceeding 200 µm in length, 5–20 μm wide, thin-walled, hyaline; copious thromboplerous hyphae with greyish content, 4–8 µm wide, sometimes tortuous. ***Pileipellis*** as an irregular ixotrichoderm of erect and semi-erect hyphae, parallel to intertwined, 2–6 μm wide, exceeding 100 µm in length and multi-articulate (pluriseptate), thin-walled, hyaline or slightly yellowish, with sometimes enlarged or clavate apex. ***Subcutis*** subregular, hyphae longer than 80 µm, 3–10(–15) μm wide, scarcely septate, thin-walled, with a pale yellowish cytoplasmic pigment and small to large crystalline deposits. ***Stipitipellis*** as an ixocutis with abundant glutinous matrix, hyphae parallel to slightly intertwined, some are very long, exceeding 200 μm, and 3–7(–10) μm wide, scarcely septate, thin- to slightly thick-walled (wall up to 1.0 (1.5) μm thick), with a pale yellowish or slightly brownish cytoplasmic pigment; presence of dense tufts of erect, semi-erect, parallel or intertwined, 2–6 µm wide hyphae immersed in gelatinous material, with a morphology similar to that of the hyphae of the pileal ixotrichoderm. ***Stipititrama*** regular to subregular, hyphae very long up to 200 µm, 5–10(–15) μm wide, scarcely septate, thin-walled, with a pale yellowish cytoplasmic pigment and copious crystalline deposits. ***Clamp-connections*** observed in all structures especially at the base of the basidia.

#### Habit, habitat, occurrence, and distribution.

Growing gregariously, in groups of a few to many basidiomata, on litter in an anthropized serpentine forest of broad-leaved trees a few hundred meters from the sea. Uncommon and localized. Thus far known only from the Dominican Republic.

#### Notes.

In the present study, five taxa are recognized within *H. subg. Hygrocybe* section *Velosae*, three of which have been previously described from Asia (*H. hypohaemacta*, *H. pellucida*, and *H. snigdha*), whereas two are formally described here. *Hygrocybe pseudohypohaemacta* is a species with numerous collections from the Caribbean Basin previously identified as *Hygrocybe aff. hypohaemacta*. In contrast, *H. variecolor* is represented by a single collection, suggesting that it is a rare species in the Dominican Republic, and the absence of corresponding sequences in GenBank supports the hypothesis that it is globally rare. Although only one ample collection comprising basidiomata at various stages of maturity is available, it is morphologically and molecularly distinctive; thus, this new species is described with the understanding that additional collections may reveal greater variation in this taxon. *Hygrocybe variecolor* is characterized by its small size, entirely slimy basidioma, striate margin, vivid scarlet-red, orange, or yellow-orange colors of the pileus, a very thick, transparent, glutinous layer on the stipe, the presence of dimorphic basidia and spores on the same basidioma, and rare pseudocystidia. The similar *H. pseudohypohaemacta* differs in habitat, smaller macro- and microbasidiospores with different *Q* values, the abundant presence of pseudocystidia, and different nrITS and nrLSU sequences.

### Hygrocybe xerophila

Taxon classificationFungiAgaricalesHygrophoraceae

Angelini, Vizzini, Dovana, M. Marchetti & Lodge sp. nov.

864F2CF2-1BAB-5DCB-BCD6-36BA407C26C2

864525

Fig. 15

#### Etymology.

The specific epithet *xerophila* is derived from the Greek xeros (dry) and phila (loving), referring to the xerophytic dry forest habitat where the species was discovered.

**Figure 15.**
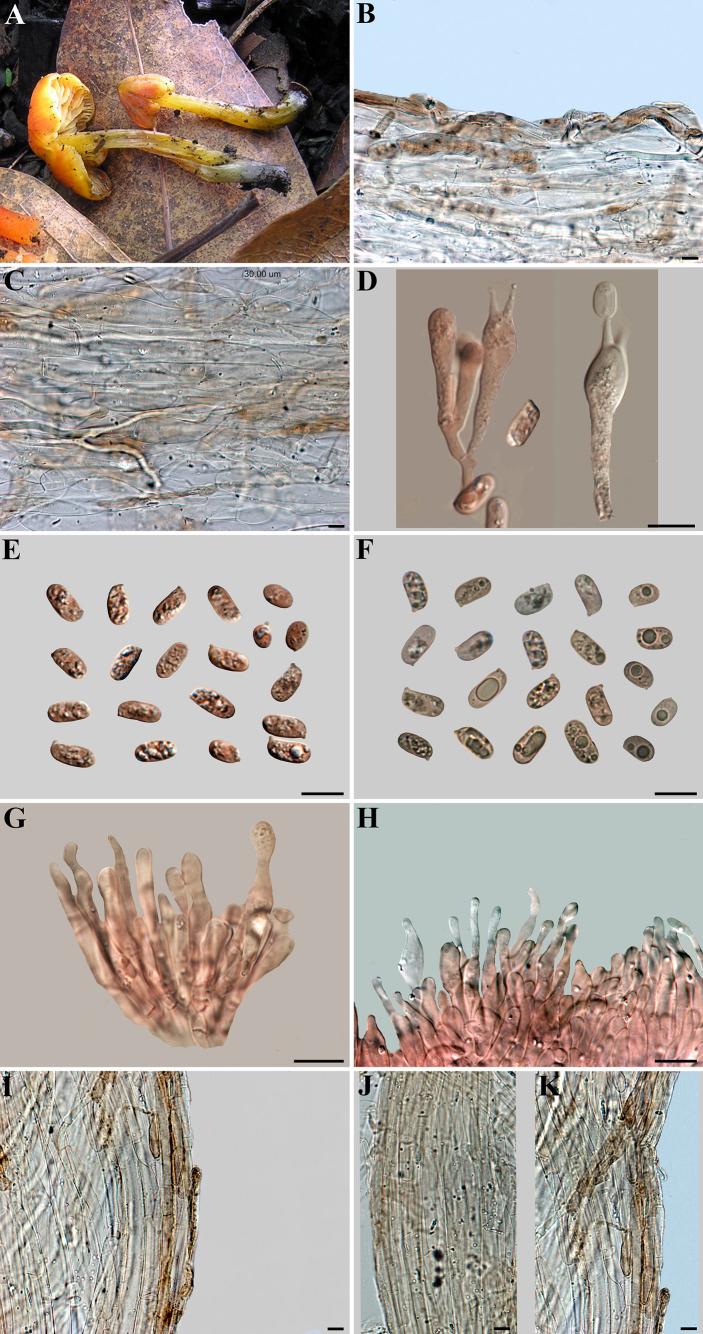
*Hygrocybe xerophila var. xerophila* (JBSD 141876, holotype). **A** Fresh basidiomata; **B** pileipellis; **C** subcutis; **D** basidia and basidiola; **E–F** basidiospores; **G–H** pseudocystidia; **I–K** stipitipellis; **J** stipititrama. **B–K** in ammoniacal Congo red. Photos by C. Angelini (**A**) and M. Marchetti (**B–K**). Scale bars: 10 μm.

#### Diagnosis.

Differing from *H. conica* in its consistently dimorphic basidia and basidiospores, the slow and faint blackening of the entire basidioma upon aging, which, on the stipe, is restricted solely to the base, and divergence in ribosomal sequences.

#### Types.

Dominican Republic, Puerto Plata, Sosúa, loc. Cemetery 19.746151, –70.540367, 23 December 2016, C. Angelini, JBSD 141876 Holotype (ANGE820 Isotype). GenBank: ITS = PZ148588.

#### Description.

***Pileus*** 15**–**25 mm diam., conical, never fully expanded, with a rounded umbo and a distinctly lobed margin; surface smooth, viscid when fresh then moist-dry, orange-yellow, light pellucid yellow and slightly striate at margin, blackening slowly with age. ***Lamellae*** adnexed, sometimes slightly emarginate, ventricose, up to 3 mm broad, thick and distant, intercalated with lamellulae, pale yellow, with irregular and concolor edge. ***Stipe*** 25–35 × 3–4 mm, cylindrical and curved, solid, yellow; surface subviscid when fresh, distinctly fibrillose-ribbed and twisted, blackening when handled or with age mostly at the base. ***Context*** thin in the pileus. ***Odor*** and ***taste*** indistinct.

***Basidiospores*** dimorphic, mostly *macrospores* (8.2–)9.4–*9.8*–10.3(–12.0) × (4.5–)4.9–*5.2*–5.6(–5.9) µm [141/2/1], *Q* = 1.46–*2.0*–2.28, *V* = 124–*149*–173 µm^3^, subcylindrical, oblong, rarely with slight median constriction, sometimes with a sub-flat adaxial side, hyaline, smooth, thin-walled, with broad and obtuse hilar appendix, guttulate (guttules greenish in “bright field” vision); *microspores* rare, (6.0–)6.6–*7.3*–7.9(–8.6) × (4.4–)4.7–*5.1*–5.4(–5.8) µm [23/2/1], *Q* = 1.1–*1.4*–1.81, *V* = 81.9–*98.9*–115.5 µm^3^_,_ ellipsoid, with evident hilar appendix, with smooth or greenish guttulous contents under optical microscopy. ***Basidia*** dimorphic, mostly *macrobasidia* (27.3–)30.9–*34.6*–38.2(–41.9) × (7.4–)7.9–*8.5*–9.0(–9.6) µm [26/2/1], *Q* = 3.6–*4.1*–4.6, *V* = 1070–*1308*–1547 µm^3^_,_ clavate to cylindrical-clavate, usually with a long, tapering pedicel, tetrasporic to bisporic, rarely monosporic, often with blackish guttulous content, sterigmata up to 8 µm long; *microbasidia* occasional (very rare) (18.9–)21.6–*26.0*–25.9(–27.0) × (4.6–)5.4–*6.1*–6.9(–7.2) µm [28/2/1], *Q* = 3.4–*3.9*–4.6, *V* = 359–*483*–607 µm^3^, cylindrical-clavate, often with blackish inner deposits, rarely with smooth, hyaline content. ***Basidiola*** subcylindrical to broadly clavate with blackish contents. ***Pleurocystidia*** and ***cheilocystidia*** absent. ***Lamellar edge*** fertile, basidia mixed with pseudocystidia. ***Pseudocystidia*** 20–40 × 4–8 µm, abundant, versiform, subcylindrical, clavate, sinuous, sublageniform, subcapitate, with median constrictions, often with appendages even 10 µm long or more, sometimes forked, with a rounded to subacute apex, often with blackish content. ***Hymenophoral trama*** regular, with more or less parallel hyphae, tubular to subfusiform, up to 100 µm long, 3–10 µm wide; rare thromboplerous hyphae with greyish contents. ***Pileipellis*** an ixocutis, hyphae mainly radial, recumbent (repent), cylindrical, generally up to 100 µm, 3–7 µm wide, containing a brownish, blackish-brown, solid to dissolved cytoplasmic pigment. ***Subcutis*** regular consisting of parallel subcylindrical hyphae, da corti a lunghi oltre 200 µm 5–15 µm wide, hyaline to pale brownish. ***Stipitipellis*** as a slightly gellified ixocutis made up of parallel subcylindrical hyphae, up to 80 µm long, 3–7 μm wide, with often enlarged or clavate terminal part, thin- to slightly thick-walled (wall up to 1 (1,5) μm thick), containing a brownish, blackish brown, solid to dilute cytoplasmic pigment. ***Stipititrama*** regular, consisting of parallel, subcylindrical hyphae, often longer than 200 µm, 5–15 µm wide, hyaline to slightly brownish; thromboplerous hyphae common, with gray, gray-brownish content. ***Clamp-connections*** observed on all hyphae especially at the base of the basidia.

#### Habit, habitat, occurrence, and distribution.

Growing gregariously, in groups of a few to many specimens, near burned ground (charcoal-pit residue) or on litter in broad-leaved forests a few kilometers from the sea. Uncommon and localized.

### Hygrocybe xerophila var. floridana

Taxon classificationFungiAgaricalesHygrophoraceae

M.E. Sm., B. Kaminsky, L. Kaminsky, Dovana, M. Marchetti, Vizzini & Lodge var. nov.

FBEA284C-12DC-5C09-9363-D43DC2DD8DE1

864527

#### Etymology.

The epithet *floridana* refers to the state of Florida (USA), where the type specimen was collected.

#### Diagnosis.

Differing from the type variety in redder pileus, larger macrospores (Q > 2), smaller microspores, geographic range and a SNP in the ITS region.

#### Types.

USA, Florida, Putnam County, Ordway-Swisher Biological Station, by Timmon’s Creek Bridge, 29.720457, –81.970066; mixed hardwood forest, *Quercus*, *Pinus* and *Carya*, 11 October 2017, B. Kaminsky, M. Caiafa, M.E. Smith, R. Stephens, A. Mujic; FLAS-F-61718 (FLAS) Holotype, (PUL) Isotype. GenBank: ITS = MH212066.

#### Description.

***Pileus*** 10–15 mm diam, broadly parabolic, not expanding, dusky orange, apparently viscid when fresh with adhering sand and debris, slight fuscous stain from handling. ***Lamellae*** adnexed, Buff Yellow, 3 mm broad, 1 mm apart, edge concolor, 1 length lamellulae inserted. ***Stipe*** 30–44 × 2.5–7 mm, flared at apex and base, yellow near base, redder in middle and apex, with dusky tints from age and handling, surface apparently viscid when fresh based on adhering sand, context solid except narrowly stuffed at apex.

***Basidiospores*** dimorphic, *macrospores* oblong, subcylindrical, cylindrical (8.0–)10.0–*11.2*–12.4(–14.5) × (3.0–)3.7–*4.1*–4.5(–5.0) µm; *Q* = 2.40–*2.73*–3.07; *microspores* ovoid, oblong or subcylindrical, (4.0–)4.5–*5.0*–5.5(–5.6) × (2.5–)2.6–*3.0*–3.4(–4.0) µm, *Q* = 1.44–*1.71*–1.98. ***Basidia*** dimorphic, microbasidia maturing before macrobasidia; *macrobasidia* broadly clavate, (24.0–)27.4–*32.2*–36.9(–40) × (6.5–)6.6–*7.7*–8.7(–9.6) µm, with large basal medallion clamp connection, 4-sterigmate; *microbasidia* (20.0–)22.3–*25.0*–27.7(–28.0) × (3.5–)3.7–*4.8*–5.9(–6.5) µm, 4-sterigmate, with small basal clamp connection. ***Pleurocystidia*** and ***cheilocystidia*** absent. ***Lamellar edge*** fertile. ***Hymenophoral trama*** regular, comprised of short parallel hyphae 2–6.4(–20) µm wide × 8–25(–80) µm long. ***Pileipellis*** a thin ixocutis over radially arranged, repent hyphae 7–16 µm wide with tapered ends and pale reddish brown contents in KOH. ***Stipitipellis*** with a thin gelatinous coating with adhering to sand over parallel hyphae 2.0–6.4 µm wide, with large medallion clamp-connections.

#### Additional specimens examined.

USA, Florida, Osceola County, DeLuca Preserve, southeast quadrat, east of US 441, near Tenant House, 27.655, –80.8803; in sugar sand, *Quercus* nearby, Pine sparse, 04/11/2021, L. Kaminsky, M.E. Smith, FLAS-F-68949 (FLAS; PUL). GenBank: ITS = OP163190.

#### Notes.

The three collections of *H. xerophila*—the type collection from the Dominican Republic and two additional collections from white sand in southernmost Florida—cluster together with an environmental sequence from the northeastern USA (Minnesota, DQ420708) and form a well-supported clade (SH-aLRT = 100, UFB = 100, BPP = 1) that is sister to *H. conica* (SH-aLRT = 96, UFB = 100, BPP = 1). The type collection of the species (JBSD 141876 from the Dominican Republic) exhibits macrospores with a mean *Q* value of 2.00, which is significantly lower than the mean *Q* values of 2.73 observed in the Florida collections (FLAS-F-61718 and FLAS-F-68949). The microspores also showed differences; in the sample from the Dominican Republic, the microspores were larger [(6.0–)6.6–7.3–7.9(–8.6) × (4.4–)4.7–5.1–5.4(–5.8) µm] than those from Florida [(4.0–)4.5–5.0–5.5(–5.6) × (2.5–)2.6–3.0–3.4(–4.0) µm]. Furthermore, the sample from the Dominican Republic contains pseudocystidia that were not observed in the Florida samples. Despite these micromorphological differences, the lack of clear separation in the Bayesian and ML analyses—the two morphotypes differ by only three nucleotides in the nrITS region—led to the treatment of these collections as phenotypic varieties of a single species. This species is characterized by its small size, basidiomata subviscid only when fresh, a lobed pileus margin, a fibrillose-ribbed and twisted stipe in the type collection, slow blackening, the presence of highly elongated dimorphic basidiospores and basidia in all collections, and growth in dry habitats. The very elongated spores, with mean *Q* values mostly ranging from 2 to 3, are notable. Halbwachs et al. (2017) observed that macrofungal spores are more elongated under depleted resources and that spores with *Q* values of 2–3 have increased dispersal fitness. A larger, caespitose *Hygrocybe* species growing in sand dunes along the coast of the southeastern USA, *H. andersonii* Cibula & N.S. Weber, has comparatively larger and more elongated basidiospores [mostly 16–19 × 3.8–5 µm, *Q* ca. 3.8–4 (Cibula and Weber 1996)]. *Hygrocybe conica* (Schaeff.) P. Kumm. *s.l*. differs from *H. xerophila* mainly in its consistently monomorphic basidia and basidiospores and the strong blackening of the entire basidioma (Kühner 1976; Kovalenko 1989; Bon 1990; Candusso 1997; Boertmann 2010, 2012; Galli 2012).

## Discussion

### Basidial/sporal dimorphism and its biological significance

The occurrence of dimorphic basidia and spores in *Hygrocybe* was initially documented by Berkeley and Broome (1871) and Petch (1917) for *H. firma* from Sri Lanka. Later, Corner (1936) provided an exhaustive ontogenetic account of basidial development for this species and *H. hypohaemacta* from Malaysia. These taxa were subsequently assigned by Heinemann (1963) to the newly established *H.* sect. *Firmae*, typified by *Hygrocybe firma* (Berk. & Broome) Singer. Members of this section exhibit a pantropical distribution and are notably absent from temperate regions (Heinemann 1966; Pegler and Fiard 1978; Pegler 1983, 1986, 1997; Lodge and Pegler 1990; Cantrell and Lodge 2001; Lodge and Ovrebo 2008; Vrinda et al. 2009; Senthilarasu et al. 2010a, 2010b; Vizzini et al. 2015). However, phylogenetic evidence provided by Lodge et al. (2014) revealed that *H.* sect. *Firmae*, as traditionally defined by morphology, is polyphyletic. Dimorphic basidia and spores appear to have evolved independently across multiple lineages, occurring within two clades of *H. subgenus Hygrocybe* (specifically sects. *Pseudofirmae* Lodge & Padamsee and *Velosae* Lodge, Ovrebo & Padamsee) and one robustly supported clade within *H. subgenus Pseudohygrocybe* Bon, representing sect. *Firmae* s.s. Consequently, Lodge et al. (2014) emended *Firmae* to include only those species lacking a partial veil and possessing basidia with a length exceeding five times that of their spores. Furthermore, the phylogenetic analysis (Fig. 1) revealed that three new species from the Dominican Republic belonging to the /conica clade—*H. xerophila* and, partially, *H. citrinonigrescens* and *H. parabolica*—also exhibit this dimorphic phenomenon. This indicates that basidial and spore dimorphism has also evolved within tropical species of *Hygrocybe* sect. *Hygrocybe*. The selection pressures driving this trait remain partially speculative, although several biological mechanisms have been proposed. Corner (1936) observed that macrobasidia arise more deeply within the subhymenium than the more superficial microbasidia. This spatial arrangement ensures that macrospores are projected farther into the interlamellar space, preventing collisions and optimizing ballistospore discharge in the typically thick, waxy gills of the genus (Corner 1936; Lodge et al. 2014). Furthermore, the production of two distinct spore sizes, along with the frequent occurrence of mono- to bisporic basidia, may represent a reproductive bet-hedging strategy (Olofsson et al. 2009; Gianella et al. 2021). In unpredictable or nutrient-poor tropical environments, larger spores likely carry greater nutrient reserves to facilitate successful colonization, whereas smaller spores maximize numerical output and the potential for long-distance wind dispersal (Lodge et al. 2014; Halbwachs and Bässler 2015). Consequently, heterospory could be an adaptation of some *Hygrocybe* species to low-nutrient and patchy habitats (cf. Pegler and Fiard 1978). This dimorphism is also intrinsically linked to aequihymeniiferous (non-coprinoid) hymenial development, a term coined by Buller (1922) to describe the uniform maturation of basidia across the lamellar surface. In these species, the maturation of basidia is staggered: macrobasidia typically develop and discharge first, followed by microbasidia, a sequence that potentially extends the total sporulation period and increases the likelihood of encountering favorable conditions for germination (Buller 1922; Corner 1936, 1947; Lodge et al. 2014). Lodge (pers. obs.) has observed staggered development of micro- and macrobasidia and spores, with microspores often maturing before macrospores in *Hygrocybe* species with basidiomata lasting a week or more [e.g., *H. batistae* Singer and *H. appalachianensis* (Hesler & A.H. Sm.) Kronaw.], which is also consistent with bet-hedging. Furthermore, macrospores might sometimes contain nuclei of opposite mating types and therefore be self-fertile, which could greatly facilitate long-distance dispersal. Successful long-distance colonization of Caribbean islands and exchanges with the mainland, followed by the radiation of allopatric species, as seen in the /appalachianensis clade, seem improbable in the absence of self-fertile macrospores.

### Global distribution and nutritional strategies of *Hygrocybe*

*Hygrocybe* species (waxcaps) exhibit a remarkably broad ecological amplitude, with habitat preferences that vary significantly among global regions. While in Europe waxcaps are typically found in extensively managed, nutrient-poor grasslands and meadows [Kovalenko 1989; Arnolds 1990; Candusso 1997; Boertmann 2010, 2012; with the exception of the *H. conica* complex and *H. quieta* (Kühner) Singer], this pattern is notably reversed not only in North America but also throughout the Tropics and Neotropics, where these species are predominantly located in forest ecosystems (Hesler and Smith 1963; Halbwachs et al. 2013a, 2013b). In these regions, waxcaps thrive in diverse woodland environments. These include disturbed evergreen seasonal forests in Mexico characterized by haplic luvisols and rendzic leptosols (Chanona-Gomez et al. 2007; Escobar-Ocampo and Ochoa-Gaona 2007), moist tropical forests in Costa Rica on humic lithosols and dystric gleysols (Gómez 1983), and pine forests in the Dominican Republic where the substrate consists of eutric and chromic cambisols (TOA 2011b). In the African tropics, such as Kenya, they are associated with humic ferralsols, nitisols, and umbric andosols (Kost 2002). Such regional differences may be attributed to the higher levels of eutrophication and acidification in Central European forests (Lorenz et al. 2006; Simpson et al. 2006), whereas many tropical and North American forests maintain the natural, low-nutrient status required by these fungi (FAO 2006). Despite the extreme diversity of associated vegetation and soil types—ranging from Neotropical broad-leaved forests to boreal conifers—a consistent prerequisite for most *Hygrocybe* species remains a marked preference for soils with low fertility, specifically low levels of mineralized phosphorus and nitrogen (Griffith et al. 2002; Boertmann 2010).

The nutritional mode of waxcaps is uncertain (Bresinsky 2008). They were previously classified as saprotrophic (e.g., Arnolds 1990; Molina et al. 1992; Rinaldi et al. 2008; Boertmann 2010, 2012; Tedersoo et al. 2010) and, more specifically, as dependent on humic compounds in the soil (Arnolds 1981). In European grasslands, it is increasingly suggested that *Hygrocybe* species establish possibly biotrophic or endophytic nutritional relationships with herbaceous plants or bryophytes. Evidence for this hypothesis includes stable isotope signatures, the failure of axenic cultivation, and the identification of *Hygrocybe* hyphae or DNA within roots, leaves, and other plant tissues (Beisenherz 2000; Griffith et al. 2002; Griffith 2004; Seitzman et al. 2011; Halbwachs et al. 2013a, 2013b; Peršoh et al. 2013; Tello et al. 2014). A taxon phylogenetically close to *Hygrocybe*, namely *Cuphophyllus virgineus* (Wulfen) Kovalenko, was shown to be a root endophyte of *Plantago lanceolata* and vertically transmitted via seeds (Tello et al. 2014). Despite their prevalence in forest environments in North America and the Tropics, Halbwachs et al. (2013a) found no evidence of ectomycorrhizal associations between *Hygrocybe* species and forest trees. However, more recent sequencing has revealed *Hygrocybe* sequences in tree roots, including those of tropical trees (Schroeder et al. 2019). *H. conica* was sequenced from pine mycorrhizae in a burned forest in Great Smoky Mountains National Park (Hughes et al. 2020), and *Gliophorus fumosus* D.J. Lodge, S. Russell & Leal Duttra was sequenced from a *Fagaceae* root in Arkansas, USA (Harries et al. 2026). Because of the reluctance of *Hygrocybe* spp. to grow in culture, a final assessment of the significance of endophytic or ectomycorrhizal occurrence in the life cycle of *Hygrocybe* will be a major challenge. This will probably require analyses of environmental metatranscriptomes against the background of basidioma-derived reference genomes. Furthermore, detailed histological investigations are also required to visualize the physical interface between the fungal mycelium and the cells of associated plants, whether they are grasses in European pastures or trees in tropical forests (Bresinsky 2008; Halbwachs et al. 2013a, 2013b; Zanne et al. 2020).

The Dominican species of *Hygrocybe subgenus Hygrocybe* are primarily characterized by their occurrence within Neotropical forest ecosystems, representing a significant ecological shift from the grassland-centric distribution typical of European waxcaps. These fungi predominantly inhabit the litter of broad-leaved forests or specialized coniferous environments, such as those dominated by *Pinus occidentalis*, showing a marked preference for nutrient-poor soils. A defining trait of these taxa is their specialized adaptation to disturbed or serpentine substrates, particularly burned ground and charcoal residues. For instance, *H. parabolica* is directly associated with charred substrates in broad-leaved forests, whereas the newly described *H. xerophila var. xerophila* frequently occurs near charcoal in xerophytic dry environments. Furthermore, *H. pseudohypohaemacta* exhibits a broad ecological tolerance for post-fire or anthropized soils, often growing gregariously near burned ground. This suggests a common evolutionary trend toward colonizing disturbed habitats within the /hypohaemacta and /conica clades. The occurrence of *Hygrocybe* species in post-fire environments is a documented yet specialized ecological phenomenon, as these fungi are generally considered fire-enhanced biotrophs rather than obligate carbonicolous taxa (Matheny et al. 2018; Raudabaugh et al. 2020). Within Great Smoky Mountains National Park, Tennessee, *H. conica* has been explicitly identified as a dominant pyrophilous responder, showing a significant increase in basidiomata production during the first two years following high-severity wildfires (Hughes et al. 2020). Soil analyses using high-throughput sequencing (HTS) in *Pinus heldreichii* forests of Montenegro revealed *H. conica* as a dominant taxon in post-fire environments (Lazarević et al. 2024). This molecular evidence highlights its role as a key pioneer species capable of rapidly colonizing high-altitude substrates disturbed by fire and altered nutrient dynamics. Furthermore, the ecological strategy of these fungi suggests that they might act as “body snatchers,” persisting within moss cushions or soil crusts until fire-induced changes in soil chemistry trigger their reproductive phase (Matheny et al. 2018; Raudabaugh et al. 2020, 2021; Karich et al. 2024).

## Supplementary Material

XML Treatment for Hygrocybe citrinonigrescens

XML Treatment for Hygrocybe jarabacoensis

XML Treatment for Hygrocybe parabolica

XML Treatment for Hygrocybe perdomoi

XML Treatment for Hygrocybe pseudohypohaemacta

XML Treatment for Hygrocybe variecolor

XML Treatment for Hygrocybe xerophila

XML Treatment for Hygrocybe xerophila var. floridana

## Additional information

### Artificial Intelligence (AI) use

The authors accept full responsibility for the content of the manuscript, including the disclosure of any use of AI.

No AI tools were used in the preparation of this manuscript.

### Adherence to national and international regulations

All the fungal strains used in this study have been legally obtained, respecting the Convention on Biological Diversity (Rio Convention).

### Author ORCIDs

F. Dovana https://orcid.org/0000-0002-8556-7692

C. Angelini https://orcid.org/0000-0002-5485-6889

D.J. Lodge https://orcid.org/0000-0003-4807-348X

M. Marchetti https://orcid.org/0000-0002-8350-6992

A. Vizzini https://orcid.org/0000-0001-8390-6446

### Data availability

The combined nrITS/nrLSU sequence alignment used for the phylogenetic analysis is deposited in Figshare (https://doi.org/10.6084/m9.figshare.32936537). The newly generated sequences are deposited in GenBank as reported in the main text.
